# Lack of hepatic apoE does not influence early Aβ deposition: observations from a new *APOE* knock-in model

**DOI:** 10.1186/s13024-019-0337-1

**Published:** 2019-10-17

**Authors:** Tien-Phat V. Huynh, Chao Wang, Ainsley C. Tran, G. Travis Tabor, Thomas E. Mahan, Caroline M. Francis, Mary Beth Finn, Rebecca Spellman, Melissa Manis, Rudolph E. Tanzi, Jason D. Ulrich, David M. Holtzman

**Affiliations:** 1 Department of Neurology, Hope Center for Neurological Disorders, Knight Alzheimer’s Disease Research Center, Washington University School of Medicine, St. Louis, MO USA; 2 Medical Scientist Training Program (MSTP), Washington University School of Medicine, St. Louis, MO USA; 3 McCance Center for Brain Health and Genetics and Aging Research Unit, Department of Neurology, Massachusetts General Hospital and Harvard Medical School, Charlestown, MA USA

**Keywords:** apoE, Apolipoprotein E, Amyloid, Aβ, apoE particle, Cre-loxP, Albumin, Mouse model

## Abstract

**Background:**

The apolipoprotein E (*APOE*) gene is the strongest genetic risk factor for late-onset Alzheimer disease (AD). ApoE is produced by both astrocytes and microglia in the brain, whereas hepatocytes produce the majority of apoE found in the periphery. Studies using *APOE* knock-in and transgenic mice have demonstrated a strong isoform-dependent effect of apoE on the accumulation of amyloid-β (Aβ) deposition in the brain in the form of both Aβ-containing amyloid plaques and cerebral amyloid angiopathy. However, the specific contributions of different apoE pools to AD pathogenesis remain unknown.

**Methods:**

We have begun to address these questions by generating new lines of *APOE* knock-in (*APOE*-KI) mice (ε2/ε2, ε3/ε3, and ε4/ε4) where the exons in the coding region of *APOE* are flanked by loxP sites, allowing for cell type-specific manipulation of gene expression. We assessed these mice both alone and after crossing them with mice with amyloid deposition in the brain. Using biochemical and histological methods. We also investigated how removal of *APOE* expression from hepatocytes affected cerebral amyloid deposition.

**Results:**

As in other *APOE* knock-in mice, apoE protein was present predominantly in astrocytes in the brain under basal conditions and was also detected in reactive microglia surrounding amyloid plaques. Primary cultured astrocytes and microglia from the *APOE*-KI mice secreted apoE in lipoprotein particles of distinct size distribution upon native gel analysis with microglial particles being substantially smaller than the HDL-like particles secreted by astrocytes. Crossing of APP/PS1 transgenic mice to the different *APOE*-KI mice recapitulated the previously described isoform-specific effect (ε4 > ε3) on amyloid plaque and Aβ accumulation. Deletion of *APOE* in hepatocytes did not alter brain apoE levels but did lead to a marked decrease in plasma apoE levels and changes in plasma lipid profile. Despite these changes in peripheral apoE and on plasma lipids, cerebral accumulation of amyloid plaques in APP/PS1 mice was not affected.

**Conclusions:**

Altogether, these new knock-in strains offer a novel and dynamic tool to study the role of *APOE* in AD pathogenesis in a spatially and temporally controlled manner.

**Electronic supplementary material:**

The online version of this article (10.1186/s13024-019-0337-1) contains supplementary material, which is available to authorized users.

## Background

Over the past 20 years, studies on apolipoprotein E (apoE) and its roles in various physiologic processes (atherosclerosis, Alzheimer disease – AD, etc..) have relied heavily on murine models that express the three main human isoforms (ε2, ε3, and ε4) under the control of the endogenous murine *Apoe* regulatory sequences [1–3]. These *APOE* knock-in mice were generated through targeted replacement strategies (referred to as *APOE-*TR mice from here onward) and have played instrumental roles in elucidating the isoform-specific differences in lipid metabolism and receptor binding affinity. In the context of AD, *APOE* modifies the risk for development of late-onset AD in an isoform-dependent manner (ε2 < ε3 < ε4, where the ε4 allele carries the highest risk) [4]. One mechanism through which *APOE* influences AD risk is through its effects on the metabolism of the amyloid-β peptide (Aβ), the main constituent of amyloid plaques found in AD patients. Indeed, crossing of transgenic mice that develop Aβ deposition in the brain (e.g. APP/PS1 or PDAPP mice that develop human-like Aβ plaques) to *APOE-*TR mice led to an isoform-dependent effect on cerebral amyloid plaque accumulation [5, 6], which is consistent with observations in humans [7]. Intriguingly, the effects of *APOE* on amyloidosis appear to be both isoform- and quantity-dependent, as reduction of apoE3 and apoE4 levels through genetic [8, 9] or pharmacologic [10] manipulations results in reduction of cerebral amyloid plaque load. While these studies shed important insights on one aspect of apoE’s role in AD pathogenesis, it remains unclear whether the effects resulted from a cell-independent or cell-autonomous mechanism.

Emerging data indicate that *APOE* not only affects AD risk, but also severity of pathology in dementia with Lewy bodies and neurodegeneration in tauopathies [11–14]. In particular, microglia-derived apoE appears to regulate the inflammatory response [11, 15–17], suggesting that the cellular source of apoE in both the brain and periphery has distinct functions in different diseases. In the brain, both astrocytes [18] and microglia [19] contribute to the pool of apoE. Additionally, apoE cannot cross the blood-brain barrier (BBB) [20], thus the pools of apoE in the central nervous system (CNS) and the periphery exist predominantly independently from one another. Some early studies in *Apoe*-deficient mice found age-dependent synaptic loss and learning deficits [21]. These deficits reflect the potential role of apoE in multiple physiologic processes responsible for maintaining brain homeostasis, including protection from oxidative damage [22, 23], maintenance of the BBB [24, 25], and cholesterol transport in the setting of synapse development [26] or neuronal injury [27]. Intriguingly, restoration of peripheral *Apoe* expression in an *Apoe* knock-out mice rescues the learning and memory deficit found in *Apoe*-deficient mice, despite exhibiting a similar degree of synaptic loss [28]. These findings suggest that both CNS and peripheral apoE (together with plasma lipids) are independent parameters that can affect neuronal function.

These and many other outstanding gaps in knowledge regarding apoE biology necessitate an experimental model where *APOE* expression can be specifically manipulated in different tissues and cell types. Here, we report the generation of an *APOE* knock-in mouse model where the various human *APOE* variants (*ε*2, *ε*3, and *ε*4) replace the endogenous murine *Apoe* locus (termed E2F, E3F, E4F mice individually, and *APOE*-KI mice collectively). Importantly, the human locus (specifically exons 2 to 4) is flanked by loxP sites that allow for the tissue-specific manipulation of *APOE* expression. We characterized the expression of apoE in the brain and brain cell types as well as the effects of *APOE* isoforms on Aβ deposition in this new model. We also investigated the effects of peripheral *APOE* knock-out (via hepatocyte-specific deletion of *APOE* expression using the *Alb* promoter) on plasma cholesterol homeostasis as well as Aβ deposition in the brain.

## Methods

### Contact for reagent and resource sharing

Further information and requests for resources and reagents should be directed to David M. Holtzman (holtzman@wustl.edu).

### Experimental model and subject details

#### Targeting construct

The targeting strategy allows the generation of a constitutive humanization of the *Apoe* gene with the various human isoforms (*APOE-ε2, APOE-ε3,* and *APOE-ε4*), as well as a conditional knock-out and a constitutive knock-out of the gene. The targeting strategy is based on Ensembl transcripts ENSMUST00000174064 (mouse, corresponding to NCBI transcript NM_009696.3) and ENST00000252486 (human, corresponding to NCBI transcript NM_000041.3). The humanized alleles express the full length human proteins, including its signal peptide. Mouse genomic sequence from the translation initiation codon in exon 2 to the termination codon in exon 4 was replaced with its human counterparts: [Cys130, Cys176] for *APOE-ε2*, [Cys130, Arg176] for *APOE-ε3,* and [Arg130, Arg176] for *APOE-ε4.* Exons 2 to 4 (~ 3.9 kb) have been flanked by LoxP sites. A polyadenylation signal (hGHpA: human Growth Hormone polyadenylation signal) has been inserted to the 3′ of the genes (downstream of the distal loxP sites) in order to prevent transcriptional read-through. Positive selection markers were flanked by FRT (Neomycin resistance – NeoR) and F3 (Puromycin resistance – PuroR) sites and inserted downstream of the proximal loxP site and upstream of the distal loxP site, respectively. The targeting vectors were generated using BAC clones from the mouse C57BL/6 J RPCI-23 and human RPCI-11 BAC libraries.

#### Generation of knock-in mice homozygous for human APOE isoforms (*APOE*-KI mice)

Targeting vectors for the various human APOE isoforms were individually transfected into the Taconic Biosciences C57BL/6 N Tac ES cell line. Homologous recombinant clones were isolated using double positive (NeoR and PuroR) and negative (Thymidine kinase – Tk) selections. The constitutive humanized/conditional knock-out alleles were obtained after in vivo Flp-mediated removal of the selection markers. The newly introduced human *APOE* gene is expressed under control of the endogenous *Apoe* promoter. The resulting strains are referred to by their specific isoform expression (E2F, E3F, and E4F), or collectively as *APOE-KI* mice. The specific DNA sequence corresponding to each isoform (see Additional file 1: Figure S1) were verified through sequencing of exon 4 by GENEWIZ. DNA was isolated from fresh-frozen brain tissues, and exon 4 was amplified using specific primers (Forward: AACAACTGACCCCGGTGG; and reverse: GCTCGAACCAGCTCTTGAGG).

#### Conditional knock-out of human *APOE* alleles

To achieve tissue-specific knock-out of the human *APOE* alleles, *APOE*-KI mice were crossed to the appropriate strain with tissue-specific expression of Cre-recombinase. Specifically, peripheral knock-out of apoE was achieved by crossing Albumin-Cre (*Alb-Cre*) mice [29] (purchased from Jackson laboratory, strain # 003574, also known as B6.Cg-Speer6-ps1^Tg(Alb-cre)21Mgn^/J) to *APOE*-KI mice for two successive generations to obtain *Alb-Cre* mice homozygous for various APOE isoforms. All mice used in this study were maintained on a C57BL/6 J background. Mice were subjected to experiments at either P7, P21, 1.5 months, or 3 months of age, per the various experimental designs. Mice were individually housed in AAALAC accredited facilities with temperature and humidity controls, and were under a 12-h light/dark cycle (lights on at 6:00 AM) cycle with free access to food and water ad libitum throughout all phases of the experiments. All animal procedures were approved by the Institutional Animal Care and Use Committee (IACUC) at Washington University, and were in agreement with the Association for Assessment and Accreditation of Laboratory Animal Care (AAALAC, WUSM).

### Method details

#### Brain extraction and preparation

At the predetermined date of brain harvesting, the appropriate mice were anesthetized with intraperitoneal pentobarbital (200 mg kg − 1), and subsequently perfused with 3 U ml − 1 heparin in cold Dulbecco’s PBS for 3 min. The brains were then dissected carefully from the skull. The right hemisphere was fixed in 4% paraformaldehyde for at least 48 h before being transferred to 30% sucrose and stored at 4 °C until they were sectioned. The left hemi-brain was dissected on an ice-cold stage into various parts (cortex, hippocampus, etc...), all of which were flash-frozen on dry ice and subsequently stored at − 80 °C until needed for biochemical analyses.

#### Histology

Following immersion in sucrose for at least 24 h, serial coronal sections (50 μm thickness) were collected from frontal cortex to caudal hippocampus (right hemisphere) using a freezing sliding microtome (ThermoFisher). Three hippocampal-containing sections (separated by 300 μm) from the right hemisphere of each brain were stained with biotinylated HJ3.4 (anti-Aβ_1–13_, mouse monoclonal antibody generated in-house, 1:500 dilution) [30] or biotinylated 3D6 antibody (anti-Aβ_1–x_) [31, 32] to visualize Aβ immunopositive plaques, as described previously. Microglia were immunostained using goat anti- IBA1 antibody (Abcam ab5076, 1:500 dilution). Astrocytes were immunostained using mouse anti-GFAP antibody (MAB3402, 1:1000 dilution). ApoE was immunostained with rabbit anti-apoE antibody (Cell signaling D719N, 1:500 dilution. All secondary antibodies were used in appropriate combinations depending on the primary antibody host, including: donkey anti-goat AF-488 (Invitrogen catalog # A-32814), donkey anti-rabbit AF-647 (Invitrogen catalog # A-31573), donkey anti-mouse AF-488 (Invitrogen catalog # A-21202), donkey anti-rabbit AF-568 (Invitrogen catalog # A-10042). All secondary antibodies were incubated at 1:500 dilution. Quantitative analysis of immunopositive staining was performed as described previously [33]. Briefly, images of immunostained sections were exported with NDP viewer (Hamamatsu Photonics). Using ImageJ software, images were converted to 8-bit grayscale, thresholded to highlight Aβ-specific staining and the percent area of a given brain region covered by thresholded staining calculated. For analyses of immunofluorescent staining (including GFAP, IBA1, apoE, X-34, and Aβ), 20X – 40X images were acquired on Nikon A1Rsi confocal microscope. Random z-stacks containing clusters of plaques were imaged, spanning approximately 30 μm of tissue in the z-plane with steps of 1.5 μm. Representative images are generated by projecting maximal intensity of each voxel on the same z-plane (using ImageJ software). All analyses were done blinded to treatment and genotype.

#### Real-time qPCR analysis

RNA was extracted from frozen cortical tissue using Trizol (Life Technologies # 15596026) and purified using the RNeasy mini kit (Qiagen # 71404). Reverse transcription was performed using a High-Capacity cDNA Reverse Transcription Kit (Life Technologies). Real-time qPCR was conducted with TaqMan primers (Life Technologies) and the TaqMan Universal PCR Master Mix (Thermo Fisher Scientific # 4304437) using the StepOnePlus machine (Applied Biosystems). Relative gene expression levels were compared using the ΔΔC_t_ method with Taqman probe for human apoE (Hs00171168_m1). Glyceraldehyde 3-phosphate dehydrogenase (GAPDH) mRNA level was used as a reference (Mm99999915_g1 Gapdh).

#### Brain homogenization

Brain cortices or hippocampi were sequentially homogenized with cold PBS and then the PBS insoluble material with 5 M guanidine buffer in the presence of 1X protease inhibitor (PI) mixture (Roche). Specifically, the frozen brain tissues were weighed on a microscale, and the ice-chilled PBS/PI buffer solution was added at a ratio of 1 ml of buffer per 100 mg tissue. The mixture were then manually homogenized with a double-ended pestle (ThermoFisher Scientific catalog # 50–256-12), until no visible chunks were seen. The samples were subsequently centrifuged at 12,000 g (4 °C) for 30 min, and the supernatants were collected as PBS-soluble homogenates. To the remaining pellet, 5 M guanidine/PI buffer were added at the same ratio (1 ml buffer per 100 mg tissue) and homogenized by sonication. The homogenates were rotated at room temperature for 1 h and subsequently centrifuged at 12,000 g (4 °C) for 30 min. The supernatants were collected as the guanidine-soluble fraction. All procedures were done on ice as much as possible unless noted otherwise.

#### Sandwich ELISA

The levels of Aβ_x-40_, Aβ_x-42_ and apoE in PBS- and guanidine-soluble brain homogenates were measured by sandwich ELISA. For apoE ELISA, HJ15.3 and HJ15.7b were used as capture and detection antibodies, respectively. For Aβ_x-40_ ELISA, HJ2 (anti-Aβ_35–40_) was used as a capture antibody and for Aβ_x-42_ ELISA, HJ7.4 (anti-Aβ_37–42_) was used as a capture antibody. HJ5.1-biotin (anti-Aβ_13–18_) [33, 34] was used as the detecting antibody for both Aβ ELISAs.

#### Primary astrocyte and microglia cultures

Mixed glial cultures were prepared from the cortex of E2F, E3F, and E4F neonatal mice (1–3 days old), similar to as previously described [35, 36]. Cortices were dissected in calcium- and magnesium-free Hanks’ Balanced Salt solution (HBSS) with careful removal of meninges. Tissue was digested in HBSS containing 0.25% trypsin and 0.2 mg/ml DNase at 37 °C for 10 min, and was dissociated by trituration in HBSS containing 0.4 mg/ml DNase. Material was filtered through a 70-μm nylon mesh, pelleted at 1000 g for 5 min, and re-suspended in glial media (DMEM + 10% FBS+ 1X Glutamax +1X Penicillin/Streptomyicin). Cells were then plated on a poly-L-lysine (PLL)-coated 10 cm dishes and then switched to glial media containing 10% L929-conditioned media (10% L929 glial media) the next day. 10% L929 glial media changes were performed every 3–4 days until cells were grown to confluence (14–16 days). The top layer of loosely attached microglia were then harvested by pipetting media over the dish ~ 10–15 times to flush off the microglia. The media containing the suspended microglia was then collected and spun down at 1000x G for 5 min at 4 °C. Microglia were then re-suspended in 10% L929 glial media and re-plated onto a 12-well plates coated with PLL. Astrocytes remaining in the 10 cm dish were detached by treating with 3 ml of 0.25% Trypsin-EDTA for 10 min at 37 °C. Seven milliliter of glial media was added to suspend the astrocytes and material was then collected and spun down at 1000 g for 5 min at 4 °C. Cells were re-suspended in glial media and re-plated onto a T75 flask coated with Geltrex. Microglia were allowed to grow for 3 days in 10% L929 glial media before being washed 2-times with sterile PBS and then switched to serum-free glial media (DMEM +1X Glutamax +1X Penicillin/Streptomyicin) containing 25 ng/ml mCSF. Astrocytes were allowed to grow for 4 days in glial media before being shaken overnight at 250 RPM at 37 °C to remove loosely attached cells from the astrocyte layer. The glial media was removed and astrocytes were then washed 2-times with sterile PBS and switched to serum-free glia media. Serum-free glial media from microglial and astrocyte cultures was collected after 48 h and stored at 4 °C for non-denaturing gel electrophoresis.

#### Non-denaturing gradient gel electrophoresis

Microglia-conditioned serum-free media and astrocyte-conditioned serum-free media samples were run on a 4–20% Tris-Glycine native non-denaturing gel at 100 V for 18 h at 4 °C. Gels were transferred to PVDF membrane at 25 V for 90 min at 4 °C and probed with an anti-ApoE antibody (HJ15.7, 1:1000; in house). ApoE immunoreactivity was detected by chemiluminescent development with ECL ultra reagents.

#### Western blot analysis

PBS-soluble brain lysates from the sequential homogenization step were analyzed for total protein concentration with a micro BCA kit (Thermo Scientific). Thirty microgram of proteins from each sample were loaded onto a NU-PAGE 4–12% Bis-Tris 15 well gel (Thermo Fisher Scientific # NP0336BOX) and the gel was run at 150 V for 1.5 h. The proteins were subsequently dry-transferred onto a PVDF membrane using the iblot2 system (Life Technologies) and blocked with 5% milk in TBS-Tween (0.05%). The membrane was incubated with anti-apoE antibody HJ15.7 [34] (or HJ15.3) and anti-β-tubulin antibodies to probe for apoE and a loading control, respectively. Donkey-anti-mouse IgG-HRP was used as secondary antibody (Santa Cruz Biotechnology # sc-2096). All blots were developed for ~ 10 s using an enhanced chemiluminescence (ECL) Ultra kit (Lumigen TMA-6) and imaged on the SynGene Imager (BioRad) at the appropriate exposure.

#### Lipid measurements

Plasma Triglyceride (Wako Diagnostics catalog # 290–63,701), HDL (HDL-Cholesterol E, Wako Diagnostics catalog # 997–01301), and Total Cholesterol (Total Cholesterol-E, Wako Diagnostics catalog # 999–02601) concentrations were measured using kits from Wako Diagnostics adapted to half-area 96-well dishes (Corning, catalog # 3690). The protocols were performed according to the manufacturer’s specifications with the following adjustments. For the triglyceride and cholesterol measurements: half volumes of the samples and standards described in the provided microplate/microtiter methods were used. For the cholesterol measurements: a standard curve with levels of 0, 25, 100, 200, 397.4, 592.2 mg/dL was used. For the HDL measurements: 20 μL sample were mixed with an equal amount of Precipitating Reagent. Due to low sample HDL concentrations, samples were loaded at twice the volume of the standard curve. As a result, the derived sample concentrations were halved to reach the true value. After adding color reagent to the wells (150 μL/well), the dishes were gently mixed and incubated at 37 °C for ~ 5 min before reading. Absorbance at 600 nm and 700 nm were measured using a Cytation 5 Imaging Reader. Analysis was performed using Gen5 software and Microsoft Excel. Absorbance at 700 nm was subtracted from that at 600 nm to correct for contaminants. Samples and standards were measured in duplicate and triplicate, respectively. Sample concentrations were derived from a linear regression fit to the standard curve. For analysis, the blank absorbance was counted as the 0 mg/dL level (it was not subtracted). Samples were subjected to ~ 1–3 freeze-thaw cycles.

### Quantification and statistical analysis

#### Statistics

**Fig. 1:**
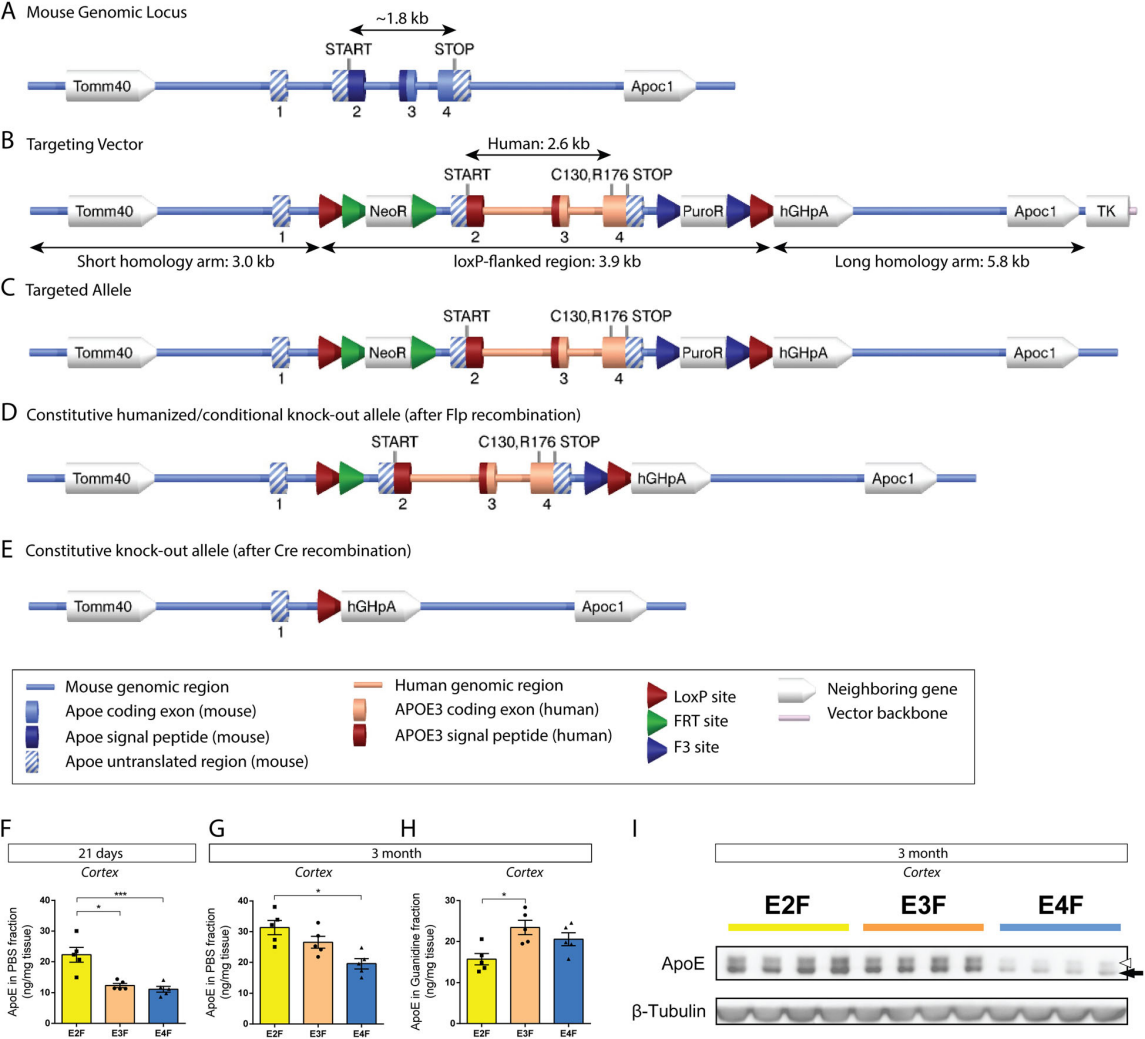
Replacement of the mouse *Apoe* gene with the human *APOE* gene in *APOE-*KI mice. **a** Genomic organization of the mouse *Apoe* gene containing exons 1–4. **b** The *APOE-ε3* targeting construct containing the 5′ and 3′ arms of mouse homology interrupted by the human *APOE-ε3* gene sequence. Exons 2 to 4 of the human sequence were flanked with loxP sites. Positive selection markers were flanked by FRT (Neomycin resistance – NeoR) and F3 (Puromycin resistance – PuroR) sites and inserted downstream of the proximal loxP site and upstream of the distal loxP site, respectively. **c** Homologous recombinant clones were isolated using double positive (NeoR and PuroR) and negative (Thymidine kinase - TK) selections. **d** The constitutive humanized/conditional knock-out alleles were achieved after in vivo Flp-mediated removal of the selection markers. The newly introduced human APOE gene is expressed under control of the endogenous *Apoe* promoter. **e** Constitutive knock-out allele is achieved when the loxP-flanked region is removed by Cre-recombinase. **f** PBS-soluble apoE protein concentration was measured in cortical brain homogenates from *APOE-*KI mice at 21 days (*p* = 0.0004, F = 15.77). **g** PBS-soluble apoE protein concentration was measured in cortical brain homogenates from *APOE-*KI mice at 3 months of age (*p* = 0.0043, F = 8.880). **h** Guanidine-soluble apoE protein concentration was also measured in cortical brain homogenates from *APOE-*KI mice at 3 months of age (*p* = 0.0144, F = 6.169). **i** The same brain homogenates were subjected to Western blot analysis for apoE using antibody HJ15.7. White arrowhead = sialylated apoE (MW ~ 35.3 kDa). Black arrow = non-sialylated apoE (MW ~ 33.6 kDa). *N* = 2 males, 2 females per genotype. *p < 0.05, ***p < 0.001. A one-way ANOVA was used to assess significance between more than two groups, and Bonferroni’s post-hoc test was used to test for differences between each of the groups. All values are reported as mean ± SEM. *N* = 5 per genotype for **f**, **g**, and **h**, with approximately equal numbers of males and females

All values are reported as mean ± SEM. A one-way ANOVA was used to assess significance between more than two groups (Fig. 1 and Additional file 1: Figure S1), and Bonferroni’s post-hoc test was used to test for differences between each of the groups. A two-way ANOVA was used to assess significance between more than two groups in the presence of additional variables (Figs. 5, 6, 7, 8, and 9), and Tukey’s post-hoc test was used to test for differences between each of the groups (Figs. 5, 6, 7, 8, and 9), with the exception of Fig. 7c – h, where Holm-Sidak multiple comparisons testing was used due to non-normal distribution of data in some groups. All statistical analyses were performed using Prism software (Graphpad). *p* < 0.05 is considered significant for all tests. No statistical analysis was used to determine sample size a priori. The sample sizes chosen are based on those used in previous studies from our laboratory. The number of samples indicates biological replicates as indicated in each of the figure legends. At most one outlier was removed per genotype via the Grubb’s test (alpha = 0.05, GraphPad QuickCalcs1).

## Results

### Design and generation of *APOE*-KI mice

In order to investigate the effects of tissue-specific *APOE* deletion, we set out to create a knock-in model that can allow for promoter-specific deletion of the *APOE* coding region under the Cre-loxP system. Three separate vector constructs with human sequences corresponding to the *ε2*, *ε3*, and *ε4* alleles of *APOE* were generated with loxP sites flanking exons 2 through 4 (Fig. 1a – e). The targeting strategy allows for the humanization of the coding region within the murine *Apoe* gene (Fig. 1a) with the various human isoforms (*APOE-ε2*, *APOE-ε3*, and *APOE-ε4*), as well as the opportunity to conditionally knock-out the coding region of the gene. Mouse genomic sequence from the translation initiation codon in exon 2 to the termination codon in exon 4 was replaced with its human counterparts: [Cys130, Cys176] for *APOE-ε2*, [Cys130, Arg176] for *APOE-ε3*, and [Arg130, Arg176] for *APOE-ε4* (Additional file 1: Figures S1b, S1c, and S1d). Exons 2 to 4 (~ 3.9 kb) are flanked by LoxP sites to allow for conditional deletion by Cre-recombinase. Homologous recombinant clones were isolated using double positive (NeoR and PuroR) and negative (Thymidine kinase - TK) selections, and the respective resistance genes were included in the targeting vector (Fig. 1b, c). The constitutive humanized/conditional knock-out alleles were achieved after in vivo Flp-mediated removal of the selection markers (Fig. 1d). In the presence of Cre-recombinase (either through directed genetic crossing with a Cre line or viral vector), constitutive knock-out of the *APOE* gene is achieved when the loxP-flanked region is removed (Fig. 1e). Of note, the chimeric locus retains all normal mouse regulatory sequences in addition to the non-coding exon 1. Exon 2 contains the translation initiation codon (Additional file 1: Figure S1a). The cleavable signal peptide is encoded within exons 2 and 3 (amino acids 1–18 – Additional file 1: Figure S1a). Due to the non-conserved cleavage sites of mouse and human signal peptides (please see alignment – Additional file 1: Figure S1a), the humanized allele expresses the full-length human *APOE* protein, including its signal peptide, rather than a fusion protein between the mouse signal peptide and the human mature protein. To verify accuracy and successful creation of the model, brain samples from all 3 lines were submitted for sequencing of exon 4 of the *APOE* locus by GENEWIZ, which confirmed the presence of human sequence and appropriate single nucleotide polymorphisms (SNPs) specific for each isoform. Further details on the specific design of the vector can be found in the methods section.

We first characterized the newly created *APOE*-KI strains by confirming the presence of human *APOE* mRNA expression in the mice with qPCR analysis (Additional file 1: Figure S1e and S1f). At 3 months of age, there was a slight, but statistically significant, elevation in *APOE* mRNA level in the hippocampus of E4F mice compared to E2F or E3F mice (Additional file 1: Figure S1e). We also assessed *APOE* mRNA levels in whole brain hemispheres. Again, *APOE* mRNA levels were similar between genotypes, although *APOE* mRNA levels were slightly lower in E2F mice compared to either E3F or E4F mice, both of which were statistically significant (Additional file 1: Figure S1f). Next, we biochemically assessed the apoE protein levels in the brains of *APOE-*KI mice at 21 days and 3 months after birth. At 21 days of age, apoE protein levels in the cortex were significantly higher in E2F mice relative to those found in E3F or E4F mice (Fig. 1f). At 3 months of age, there was no difference in PBS-soluble apoE protein concentration between E2F and E3F mice, while E4F mice had significantly lower levels relative to E2F mice (Fig. 1g). We also measured guanidine-soluble apoE and found E3F mice to have significantly higher levels than E2F mice (Fig. 1h). Lower apoE protein levels in 3-month-old E4F mice were also seen via Western blot analysis of the same brain lysates utilizing HJ15.7 as the detecting antibody (Fig. 1i). This latter finding was replicated using another anti-apoE antibody (Additional file 1: Figure S1g). These data demonstrated that the newly generated *APOE*-KI mice expressing different apoE isoforms have similar levels of human *APOE* mRNA in the brain. Some differences in protein levels (higher apoE2, lower apoE4) are likely secondary to differences in protein stability, half-life, and metabolism, as has been seen in previous *APOE* knock-in mice [6, 37–44].

### Human *APOE* is expressed in astrocytes and microglia in *APOE*-KI mice

**Fig. 2:**
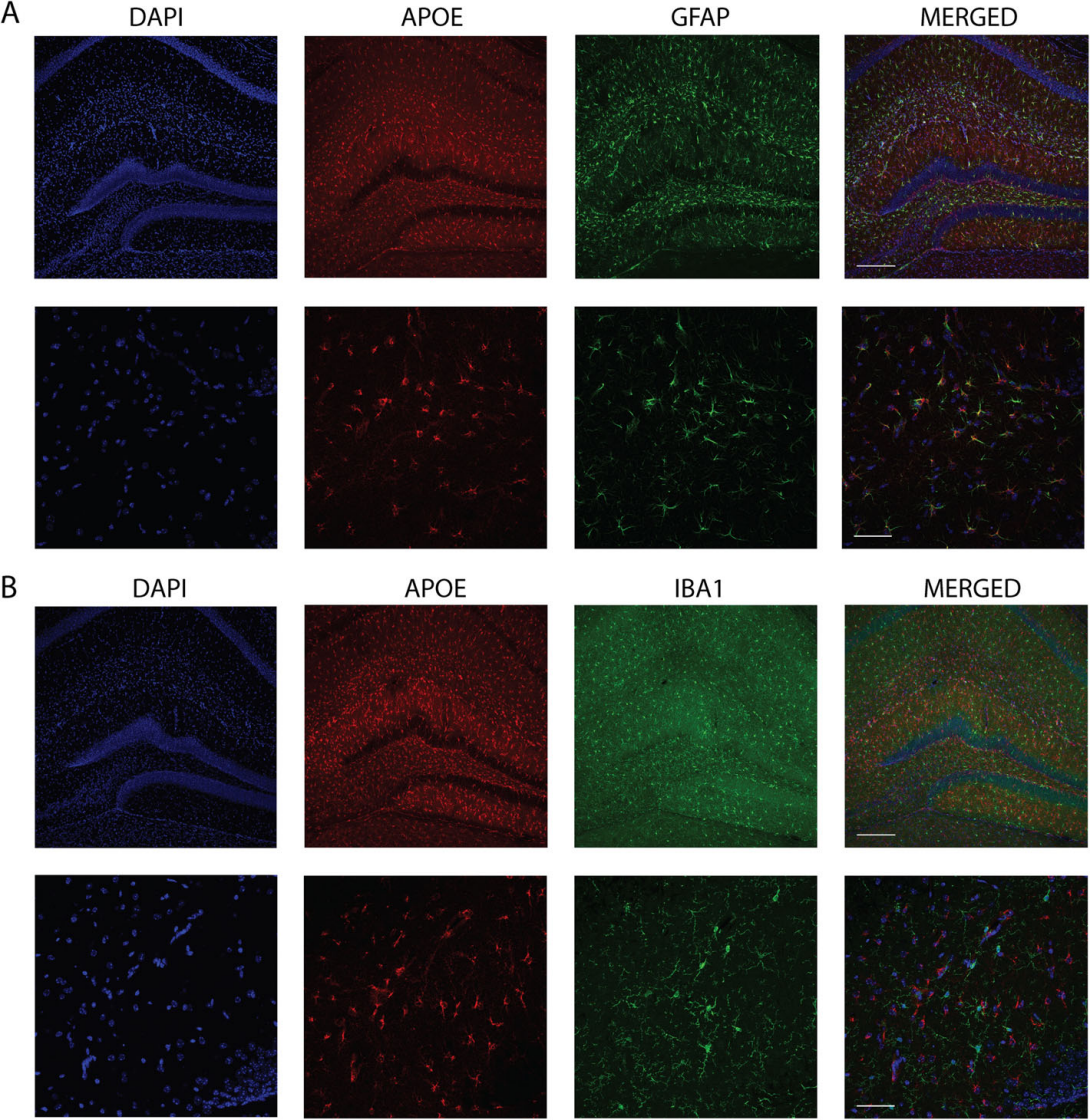
Human *APOE* is expressed in astrocytes in *APOE-*KI mice. **a** Brain sections from *APOE*-KI mice were co-stained for nuclei (DAPI), apoE, and GFAP. Multiple foci of ApoE/GFAP co-localization can be seen at high magnification (bottom panels). **b** Brain sections from *APOE-*KI mice were co-stained for nuclei (DAPI), apoE, and IBA1. No overlap of apoE and IBA1 staining was observed. Scale bars = 200 μm (top panels) and 50 μm (bottom panels). Images are from E4F mice, and are representative of at least 3 random cortical areas from 3 biological replicates. There were no appreciable qualitative differences between E2F, E3F, and E4F samples

The majority of apoE molecules in the CNS are synthesized by astrocytes [18], with a small portion coming from microglia [19]. We further characterized the expression pattern of apoE in the brain of *APOE-*KI mice by co-staining for apoE and traditional markers for astrocytes as well as microglia. We confirmed the presence of apoE protein in astrocytes by co-staining for apoE and the astrocytic marker GFAP (Fig. 2a). There was some apoE staining in cells with the morphology of astrocytes that were GFAP-negative. We also assessed microglia for the presence of apoE protein by co-staining for the microglial marker IBA1, however, we did not observe significant overlap of apoE and IBA1 signal (Fig. 2b). For simplicity, only representative images from E4F mice are shown, as similar findings were found in E2F and E3F mice.

**Fig. 3:**
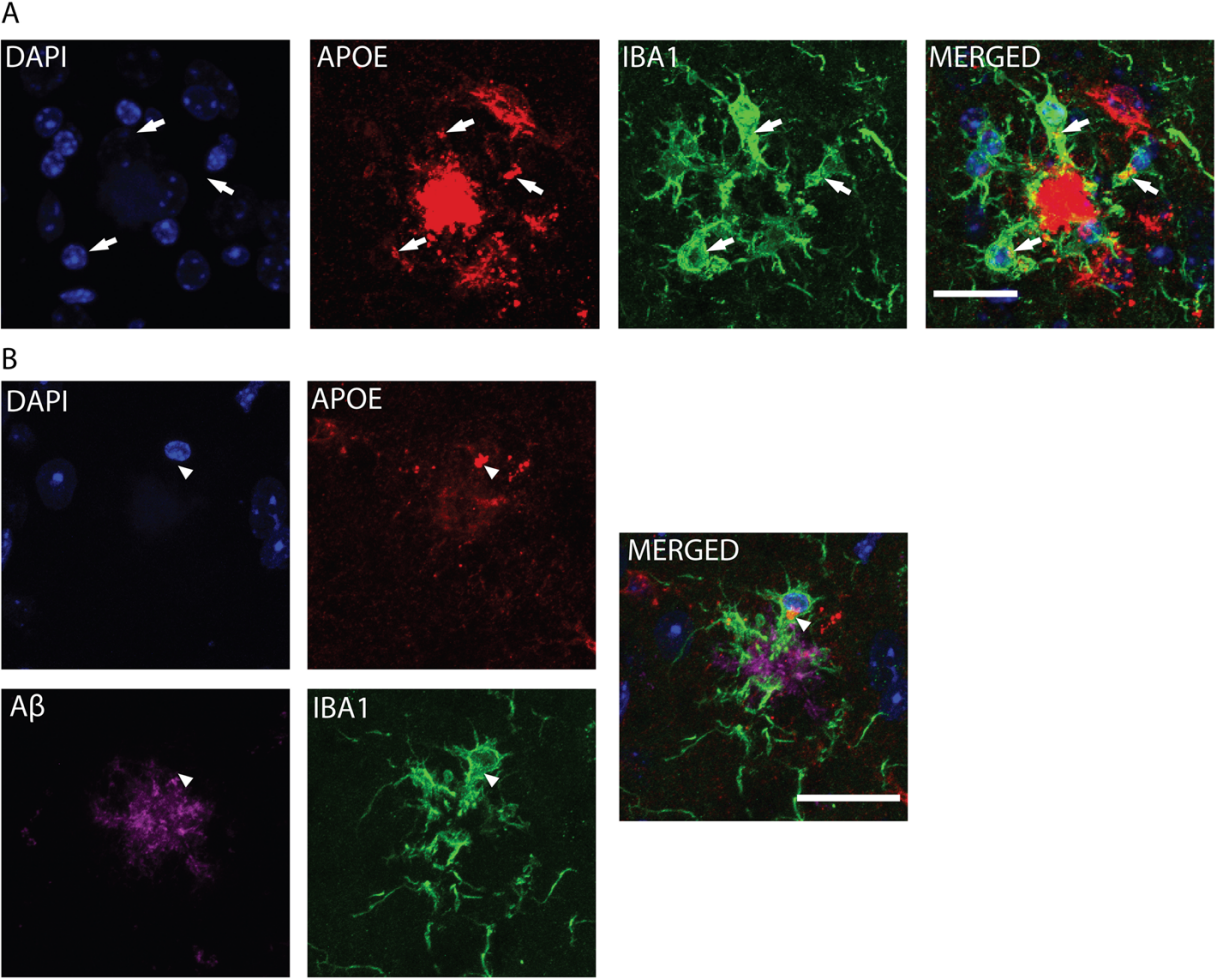
Microglial *APOE* expression in APP/PS1/EKI mice. **a** Brain sections from APP/PS1/E4F mice were co-stained for nuclei (DAPI), apoE, and IBA1. Multiple foci of apoE/ IBA1 co-localization can be seen (arrows). Scale bar = 20 μm **b** Brain sections from APP/PS1/E4F mice were co-stained with DAPI, apoE, Aβ, and IBA1. ApoE is co-localized with IBA1 (arrowhead). Scale bar = 25 μm. Images are representative of at least 3 random cortical areas from 3 biological replicates. There were no appreciable qualitative differences between APP/PS1/E2F, APP/PS1/E3F, and APP/PS1/E4F samples

ApoE’s role in AD pathogenesis was first recognized when apoE was found to co-localize with amyloid plaques, specifically at the center (i.e. the “core”) of mature, fibrillar amyloid plaques [45, 46]. ApoE expression is low in microglia under basal, homeostatic conditions, but is strongly up-regulated in the setting of various neurodegenerative insults [17, 47–49]. Thus, we investigated whether apoE can be found in microglia in the setting of amyloidosis, specifically in the APP/PS1–21 model which develops Aβ deposition in amyloid plaques beginning at 6–8 weeks of age [50]. APP/PS1–21 mice were crossed with *APOE*-KI mice for two successive generations and the brain sections from 4-month-old APP/PS1–21 mice homozygous for human *APOE* alleles (ε2/ε2, ε3/ε3, or ε4/ε4) were subjected to immunohistochemical analysis. Qualitative assessment of the staining pattern showed localization of apoE in the center of plaques, and significant co-localization with IBA1 in surrounding microglia, suggesting microglial expression of apoE (Fig. 3a, b). We made similar observations in APP/PS1–21 mice expressing *APOE-ε2* and *APOE-ε3* (data not shown)*.* These histological observations confirm the presence of apoE in astrocytes and microglia, which is consistent with previous studies, and highlight the validity of our model system.

### Qualitative assessment of microglia and astrocyte-derived apoE particles

Most of the biologically active apoE exists in the brain in lipidated HDL-like particles and alterations in the lipidation state of apoE have been shown to drastically affect Aβ accumulation in models of Aβ amyloidosis [51–55]. Thus, we investigated whether apoE particles from astrocytes and microglia are comparable in size, which is associated with the amount of lipidation.

**Fig. 4:**
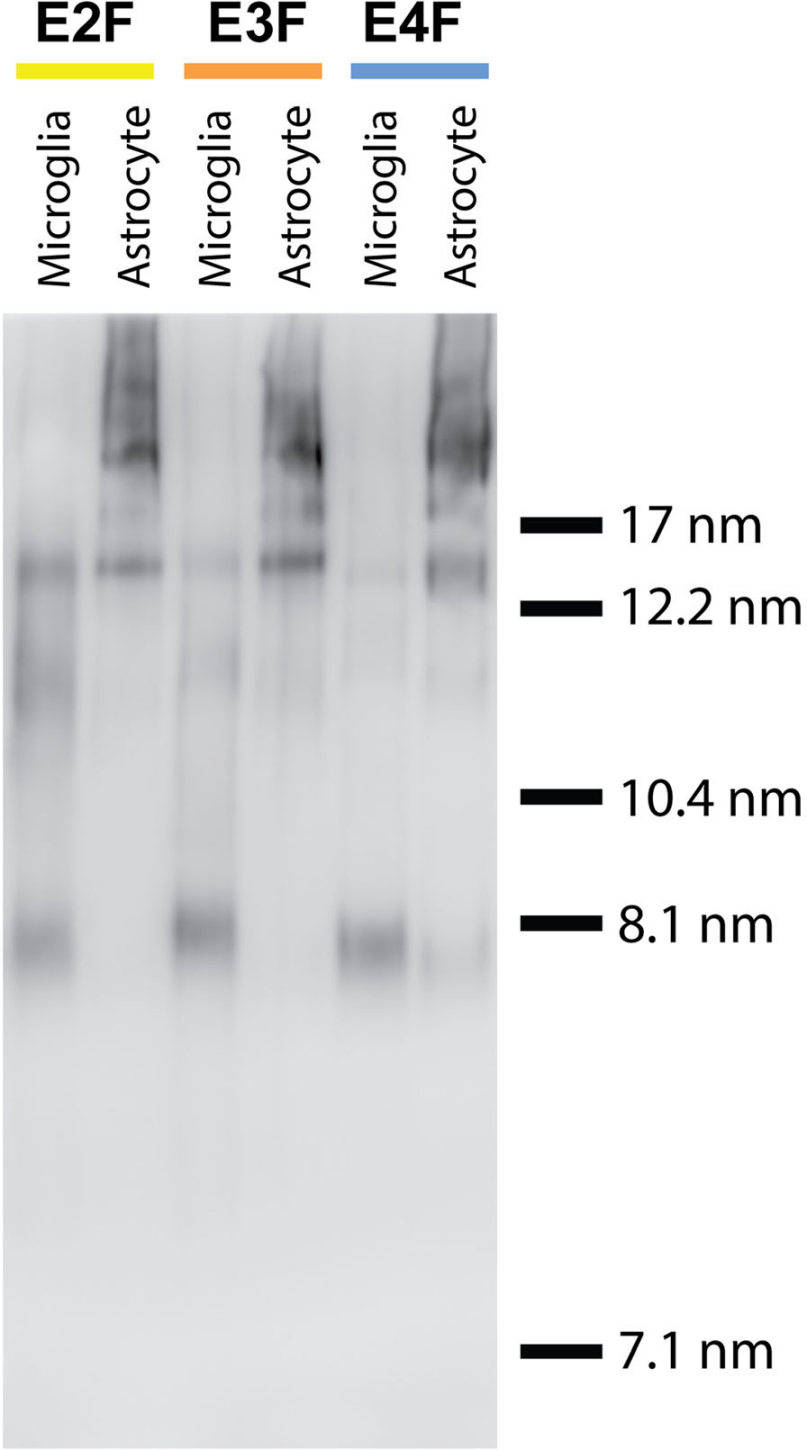
Qualitative assessment of microglia and astrocyte-derived apoE particles. Conditioned media samples from E2F, E3F, and E4F-derived primary cultures enriched for microglia and astrocyte were subjected to non-denaturing 4–20% Tris-glycine gradient gel electrophoresis followed by Western blotting. Approximate hydrated radius of marker proteins, run on the same gel, are shown for comparative purposes. Data shown are representative of 3 independent cultures from different cohorts of mice

To assess how apoE lipoprotein particles derived from microglia compare to those derived from astrocytes, conditioned media samples from primary microglial and astrocyte cultures derived from post-natal day 1–3 E2F, E3F, and E4F pups were subjected to non-denaturing gradient gel electrophoresis (NDGGE) followed by Western blotting. ApoE-containing lipoprotein particles from astrocyte-conditioned media for all three *APOE* isoforms were > 12 nm in diameter, consistent with what has been reported previously (Fig. 4) [35, 36]. While the E2F and E3F astrocyte-derived particles showed little to no particles that were < 12 nm in size, E4F astrocytes did appear to produce a small, but notable, amount of approximately 8 nm-sized particles (Fig. 4). Microglia-conditioned media contained apoE particles that were overall much smaller than the astrocyte-derived particles. For E3F and E4F microglia, the majority of particles produced were about 8 nm in size with a small amount of particles 10–17 nm in size (Fig. 4). However, for E2F microglia there did appear to be a shift in the relative amount of 10–15 nm-sized particles versus 8-nm-sized particles. While E2F microglia did produce a considerable amount of ~ 8 nm-sized particles, more 10–15-nm-sized particles were present than what was seen for E3F and E4F microglia. As larger particles contain greater amounts of cholesterol and phospholipid, these findings suggest that microglia secrete poorly lipidated apoE relative to the larger HDL-like lipoproteins secreted by astrocytes. These results highlight the need for future studies to more closely examine the properties of these apoE-containing particles and whether they also differ in their normal function as well as in pathological states.

### *APOE* isoform-dependent effect on Aβ accumulation in APP/PS1/EKI mice

While *APOE* may influence AD pathogenesis in several ways, one of the major mechanisms is via its effect on Aβ accumulation in the brain, specifically on Aβ seeding and clearance. As previous *APOE* knock-in mice have been shown to influence Aβ deposition in an isoform-dependent fashion [5, 6, 8, 9, 43, 56, 57], we wanted to assess the effects of the major human *APOE* isoforms in the new *APOE*-KI model. Specifically, we investigated the effect of different human *APOE* alleles on Aβ accumulation in APP/PS1–21 transgenic mice. In this model, overexpression of the amyloid precursor protein (APP) in neurons leads to cerebral accumulation of Aβ-containing plaques that resemble those found in AD brains [50]. We crossed APP/PS1–21 mice on a C57BL/6 background with either E2F, E3F, or E4F mice on a C57BL/6 background to obtain APP/PS1–21 transgenic mice on an *APOE-ε2*, *ε3*, or *ε4* background (APP/PS1/E2F, APP/PS1/E3F, and APP/PS1/E4F mice, respectively) that do not express murine *Apoe* (collectively referred to as APP/PS1/EKI mice). We first measured the amount of PBS-soluble apoE in the cortex of APP/PS1/EKI mice via ELISA (Fig. 6a) and found overall apoE protein levels to be similar to those seen in *APOE*-KI mice. ApoE protein concentration in the cortex of female APP/PS1/E4F mice was slightly, albeit statistically significantly, higher than those found in female APP/PS1/E3F mice.

**Fig. 5:**
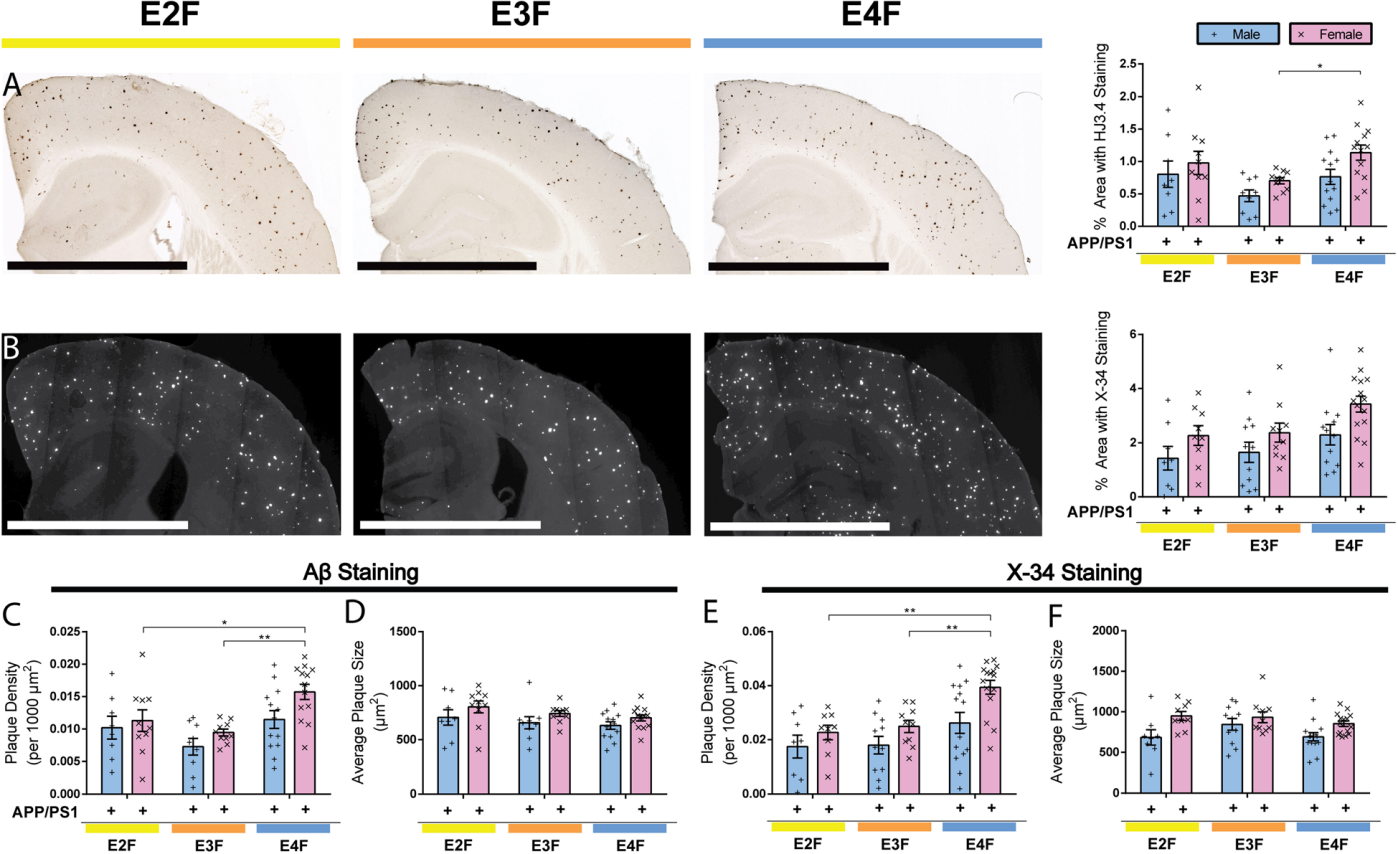
ApoE isoforms and sex differentially influence Aβ plaque deposition in APP/PS1/EKI mice. **a** Brain sections from 4-month-old APP/PS1/EKI mice were immunostained with anti-Aβ antibody (HJ3.4-biotin) and the extent of Aβ deposition in the cortex quantified. There was a significant effect of sex (F_1,57_ = 5.792, *p* = 0.0194) and apoE isoform (F_2,57_ = 4.356, *p* = 0.0174) but no interaction (F_2,57_ = 0.3269, *p* = 0.7225). Post hoc analysis comparing apoE isoform within each sex found a statistically significant increase in Aβ deposition in female apoE4-expressing mice compared to apoE3 (*p* = 0.0466), APP/PS1/E2F = 8 males, 10 female; APP/PS1/E3F = 9 males, 10 females; APP/PS1/E4F = 13 males, 13 females. Scale bar = 1 mm. **b** Brain sections from 4-month-old APP/PS1/EKI mice were stained with X-34 dye that recognizes only fibrillar plaques and the cortical area stained by X-34 was quantified. There was a significant effect of sex (F_1,59_ = 9.008, *p* = 0.0039) and apoE isoform (F_2,59_ = 4.838, *p* = 0.0113) but no interaction (F_2,59_ = 0.1898, *p* = 0.8276). Post hoc analysis comparing apoE isoform within each sex did not find a statistically significant difference, although there was a trend towards elevated X-34 staining in apoE4-expressing female mice compared to apoE2 (*p* = 0.0602) and apoE3 (0.0830), APP/PS1/E2F = 8 males, 9 females; APP/PS1/E3F = 11 males, 10 females; APP/PS1/E4F = 12 males, 15 females. Scale bar = 1 mm. **c** The density of plaques from 4-month old APP/PS1/EKI mice was calculated. There was a significant effect of sex (F_1,57_ = 5.101, *p* = 0.0278) and apoE isoform (F_2,57_ = 8.8085, *p* = 0.0008) but no interaction (F_2,57_ = 0.7514, *p* = 0.4763). Post hoc analysis comparing apoE isoform within each sex found a statistically significant increase in Aβ plaque density in female apoE4-expressing mice compared to apoE3 (*p* = 0.0032) or apoE2 (*p* = 0.0484), APP/PS1/E2F = 8 males, 10 females; APP/PS1/E3F = 9 males, 10 females; APP/PS1/E4F = 13 males, 13 females. **d** The average plaque size was quantified. There was a significant effect of sex (F_1,57_ = 5.410, *p* = 0.0236), but not of apoE isoform (F_2,57_ = 2.202, *p* = 0.1420) with no interaction (F_2,57_ = 0.05053, *p* = 0.9508), APP/PS1/E2F = 8 males, 10 females; APP/PS1/E3F = 9 males, 10 females; APP/PS1/E4F = 13 males, 13 females. **e** The density of X-34+ plaques was quantified. There was a significant effect of sex (F_1,61_ = 9.527, *p* = 0.0030) and apoE isoform (F_2,61_ = 9.941, *p* = 0.0002) but no interaction (F_2,61_ = 0.8835, *p* = 0.4186). Post hoc analysis comparing apoE isoforms within each sex found a statistically significant increase in plaque density in female apoE4-expressing mice compared to apoE3 (*p* = 0.0053), or apoE2 (*p* = 0.0016), APP/PS1/E2F = 8 males, 9 females; APP/PS1/E3F = 11 males, 10 females; APP/PS1/E4F = 14 males, 15 females. **f** The average X-34+ plaque size was quantified. There was a significant effect of sex (F_1,61_ = 11.97, *p* = 0.0010), but not apoE isoform (F_2,61_ = 2.094, *p* = 0.1320) with no interaction (F_2,61_ = 0.9353, *p* = 0.3980). All statistics were performed using a 2-way ANOVA followed by Tukey post-hoc test. *p < 0.05, **p < 0.01. All values are reported as mean ± SEM

Since the APP/PS1–21 mice have visible neocortical plaque deposits beginning around 2 months of age [50], we assessed plaque accumulation in APP/PS1/EKI mice at 4 months of age, when sufficient plaques are present in the neocortex to allow for quantitative assessments. We first examined cerebral plaque load histologically by staining brain sections with an anti-Aβ antibody HJ3.4b (Fig. 5a). Quantitative analysis of the area covered by HJ3.4b staining in the cortex showed independent, but significant, effects of sex and *APOE* isoform. Post hoc analysis comparing *APOE* genotype within each sex found a significant increase in Aβ deposition in female apoE4-expressing mice compared to apoE3. There were no significant differences in Aβ burden between APP/PS1/E2F mice (males or females) and either of the other *APOE* genotypes. We next quantified the area covered by X-34 staining, which detects fibrillar amyloid plaques (Fig. 5b). Again, there were significant and independent effects of sex and *APOE* genotype. Post hoc analysis comparing apoE isoform within each sex did not find a statistically significant difference, although there was a trend towards elevated X-34 staining in apoE4-expressing female mice compared to apoE2 and apoE3. The relative differences in cortical burden of Aβ and X-34 pathology between different isoforms are very similar to what we observed when APP/PS1–21 mice were crossed to *APOE-*TR mice (Additional file 2: Figure S2a and S2c) and in our published data [8]. Interestingly, there was no effect of sex on Aβ pathology in this latter model (Additional file 2: Figure S2b and S2d).

To characterize the factors that account for the differences in Aβ pathology, we assessed the plaque density and average plaque size in APP/PS1/EKI mice. For Aβ staining, there was a significant effect of sex and *APOE* genotype, but no interaction. Post hoc analysis showed an increase in plaque density in female APP/PS1/E4F compared to APP/PS1/E3F mice (Fig. 5c). There was a significant effect of sex, but not of *APOE* genotype, on average plaque size (Fig. 5d). For X-34 staining, there was a significant effect of sex and *APOE* genotype (but no interaction) on plaque density. Post hoc analysis comparing *APOE* genotype within each sex found a significant increase in plaque density in female APP/PS1/E4F mice compared to APP/PS1/E3F or APP/PS1/E2F mice (Fig. 5e). There was a significant effect of sex, but not *APOE* genotype on average X-34 plaque size. No differences were detected on post hoc analysis between any subgroup (Fig. 5f).

**Fig. 6:**
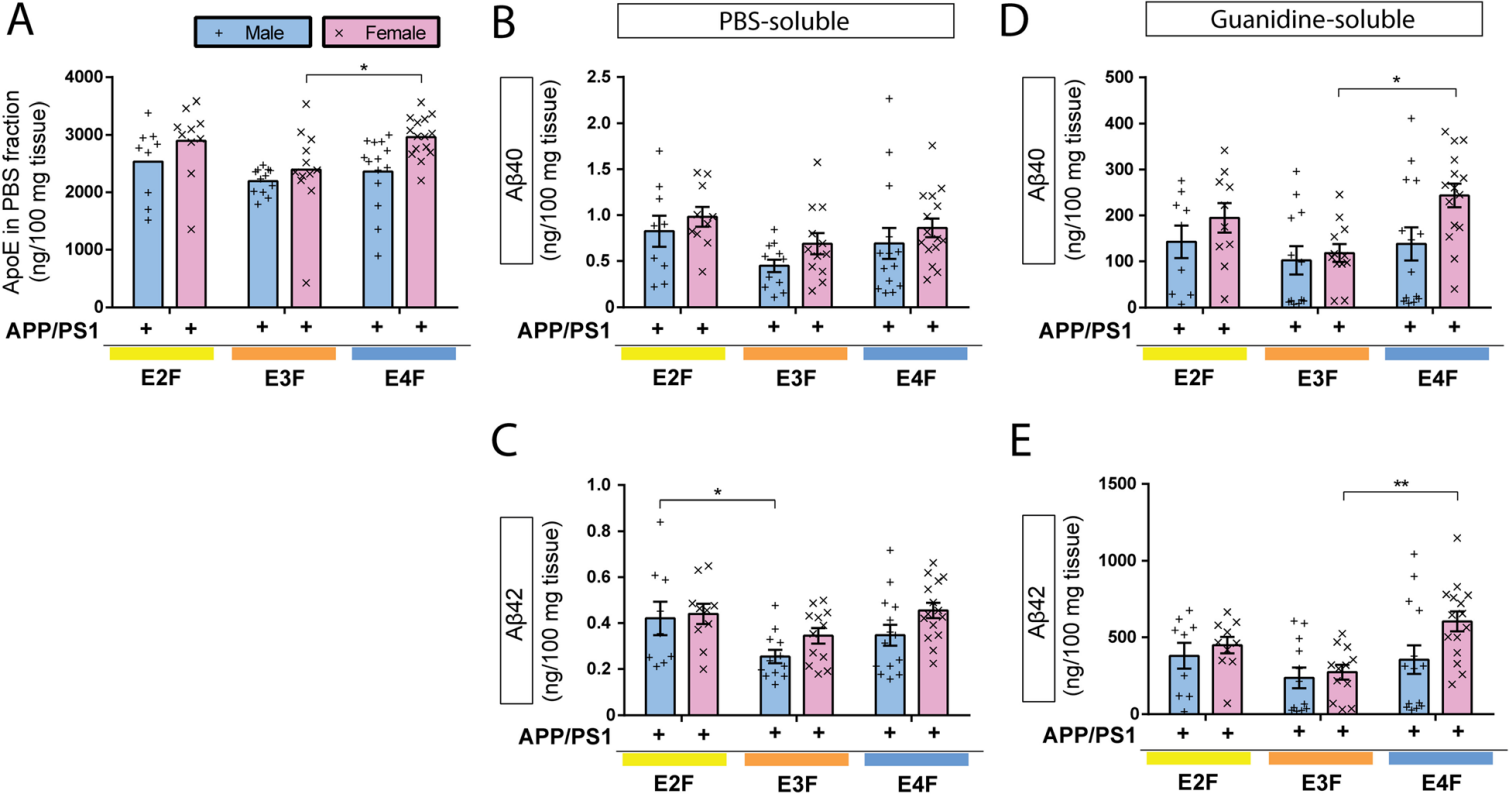
ApoE isoforms and sex differentially influence Aβ accumulation in APP/PS1/EKI mice. Cortical tissues from 4-month-old APP/PS1-EKI mice were sequentially homogenized in PBS and 5 M guanidine-HCl buffer. **a** The amount of PBS-soluble apoE was quantified by ELISA. There was a significant effect of sex (F_1,66_ = 8.373, *p* = 0.0052) and apoE isoform (F_2,66_ = 3.911, *p* = 0.0248) but no interaction (F_2,66_ = 0.8546, *p* = 0.4301). Post hoc analysis comparing apoE isoform within each sex identified a statistically significant increase in apoE levels in female apoE4-expressing compared to apoE3 (*p* = 0.0293), **b** The amount of PBS-soluble Aβ_40_ was quantified by ELISA. There was a significant effect of apoE genotype (F_2,66_ = 3.204, *p* = 0.0470), and a trend towards a significant effect of sex (F_1,66_ = 3.203, *p* = 0.0781), with no interaction (F_2,66_ = 0.06043, *p* = 0.9414). Post hoc analysis comparing apoE isoforms within each sex did not identify statistically significant pair-wise differences. **c** The amount of PBS-soluble Aβ_42_ was quantified by ELISA. There was a significant effect of sex (F_1, 66_ = 4.246, *p* = 0.0433) and apoE isoform (F_2,66_ = 4.943, *p* = 0.0100) without interaction (F_2,66_ = 0.5411, *p* = 0.5847). Post hoc analysis comparing apoE isoform within each sex identified a statistically significant increase in Aβ_42_ levels in male apoE2-expressing mice compared to apoE3 (*p* = 0.0338). **d** The amount of guanidine-soluble Aβ_40_ was quantified by ELISA. There was a significant effect of sex (F_1, 66_ = 5.240, *p* = 0.0253) and apoE isoform (F_2,66_ = 3.953, *p* = 0.0239) without interaction (F_2,66_ = 1.211, *p* = 0.3044). Post hoc analysis comparing apoE isoforms within each sex identified a statistically significant increase of Aβ_40_ in female apoE4-expressing mice compared to apoE3 (*p* = 0.0086). **e** The amount of guanidine-soluble Aβ_42_ was quantified by ELISA. There was a trend towards a significant effect of sex (F_1, 66_ = 3.915, *p* = 0.0520) and a significant effect of apoE isoform (F_2,66_ = 5.476, *p* = 0.0063) without interaction (F_2,66_ = 1.384, *p* = 0.2578). Post hoc analysis comparing apoE isoform within each sex identified a statistically significant increase in Aβ_42_ levels in female apoE4-expressing mice compared to apoE3 (*p* = 0.0030). 2-way ANOVA, Tukey’s post-hoc test, APP/PS1/E2F = 9 males, 10 females; APP/PS1/E3F = 12 males, 12 females; APP/PS1/E4F = 14 males, 15 females. Data plotted as mean ± SEM. * *p* < 0.05, ** *p* < 0.01

To further assess the total amount of Aβ accumulation, we measured the amount of PBS-soluble and PBS-insoluble (guanidine fraction) Aβ_40_ and Aβ_42_ in the cortex of APP/PS1/EKI mice. We observed a significant increase in PBS-soluble Aβ_40_ and Aβ_42_ in APP/PS1/E2F mice compared to APP/PS1/E3F mice (Fig. 6b and c) and significantly higher levels of Aβ_42_ in APP/PS1/E4F compared to APP/PS1/E3F mice (Fig. 6c). In the guanidine fraction (PBS-insoluble fraction) where the majority of Aβ accumulates, we detected a significant increase (~ 2-fold) in insoluble Aβ_42_ in APP/PS1/E4F mice relative to APP/PS1/E3F mice (Fig. 6d and e). There were no statistically significant differences between the other groups.

Our findings are consistent with previous studies where *APOE-*TR mice were crossed to various models of amyloidosis [58] and recapitulate (at least in part) the allele-dependent effect of *APOE* on amyloid deposition found in humans.

### Plasma lipid alterations in mice lacking liver-derived apoE

Previous studies from our lab and others showed that complete ablation [59–63] or reduction [8–10] of apoE levels results in a decrease of Aβ pathology, particularly a marked reduction of fibrillar Aβ in the brain. However, it was difficult to assess the contribution of peripheral apoE ablation to phenotypes found in the brain in complete knock-out models. Thus, it remains unclear whether peripherally-derived apoE (or lack thereof) exerts any effect on cerebral amyloid pathology when apoE is still present in the brain. To this end, we took advantage of the newly created *APOE*-KI lines and investigated whether a lack of the main source of peripheral apoE in the blood, (i.e. the liver), can influence Aβ pathology in the brain.

**Fig. 7:**
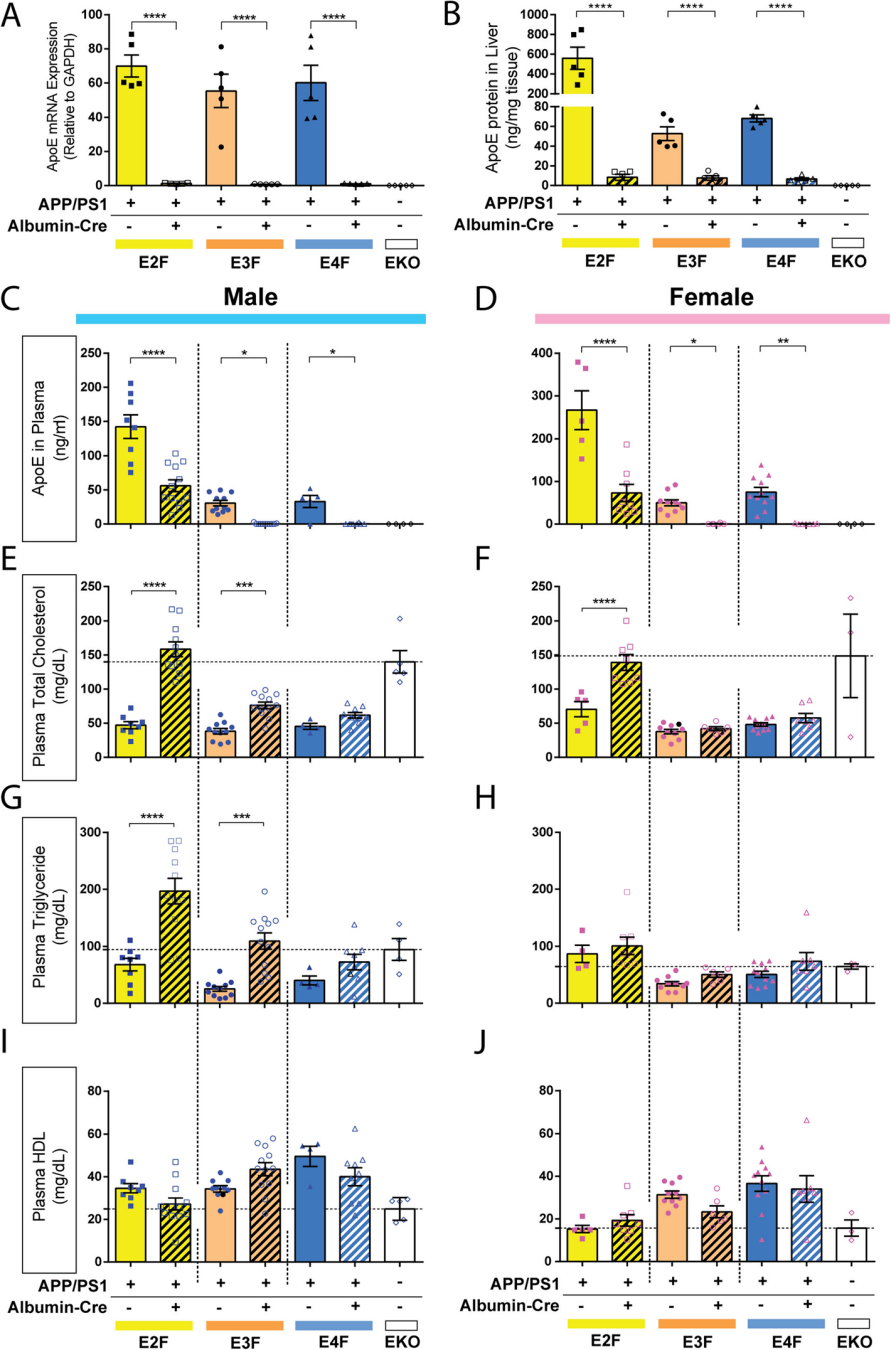
Plasma lipid alterations in APP/PS1/EKI mice lacking hepatocyte-derived apoE. **a** *APOE* mRNA levels in liver tissues from 4-month-old APP/PS1-EKI mice were analyzed by qPCR (*p* < 0.0001, F = 31.60). Post hoc analysis showed a significant difference between *Cre*^+/−^ and *Cre*^−/−^ mice in all *APOE* genotypes (*p* < 0.0001 for all). **b** PBS-soluble apoE protein concentration was measured in liver homogenates from the same cohort of mice (*p* < 0.0001, F = 23.03) Post hoc analysis showed a significant difference between *Cre*^+/−^ and *Cre*^−/−^ mice in all *APOE* genotypes (*p* < 0.0001 for all). Data for **a** and **b** were analyzed by one-way ANOVA, followed by Bonferroni’s post-hoc test for differences between each of the groups. *N* = 3 males, 2 females in each group. **c** Plasma apoE levels in male mice were measured by ELISA. There was a significant effect of apoE isoform (F_2,49_ = 70.97, *p* < 0.0001) and *Cre* expression (F_1,49_ = 51.79, *p* < 0.0001) with a significant interaction (F_2,49_ = 7.748, *p* = 0.0012). Post hoc pair-wise comparisons between *Cre*^+/−^ and *Cre*^−/−^ groups found *Cre* expression significantly decreased plasma apoE levels across apoE isoforms. (apoE2, *p* < 0.0001; apoE3, *p* = 0.0112; apoE4, *p* = 0.0232). (n; E2F: 8 *Cre*^−/−^, 12 *Cre*^+/−^; E3F: 11 *Cre*^−/−^, 12 *Cre*^+/−^; E4F: 5 *Cre*^−/−^, 8 *Cre*^+/−^). **d** Plasma apoE levels in female mice were measured by ELISA. There was a significant effect of apoE isoform (F_1,41_ = 43.03, *p* < 0.0001) and *Cre* expression (F1,41 = 61.81, *p* < 0.0001) with a significant interaction (F2,41 = 9.954, *p* = 0.0003). Post hoc pair-wise comparisons between *Cre*^+/−^ and *Cre*^−/−^ groups found *Cre* expression significantly decreased plasma apoE levels across apoE isoforms. (apoE2, *p* < 0.0001; apoE3, *p* = 0.0372; apoE4, *p* = 0.0025). (n; E2F: 5 *Cre*^−/−^, 8 *Cre*^+/−^; E3F: 11 *Cre*^−/−^, 6 *Cre*^+/−^; E4F: 11 *Cre*^−/−^, 7 *Cre*^+/−^) **e** Total cholesterol in male mice was quantified. There was a significant effect of apoE isoform (F_2,49_ = 31.14, *p* < 0.0001) and *Cre* expression (F_1,49_ = 86.12, *p* < 0.0001) and a significant interaction (F_2,49_ = 24.10, *p* < 0.0001). Post hoc pair-wise comparisons between *Cre*^+/−^ and *Cre*^−/−^ groups found *Cre* expression significantly increased plasma cholesterol levels in mice expressing apoE2 (*p* < 0.0001) or apoE3 (*p* = 0.0001), but not apoE4 (*p* = 0.1971). (n; E2F: 8 *Cre*^−/−^, 12 *Cre*^+/−^; E3F: 11 *Cre*^−/−^, 12 *Cre*^+/−^; E4F: 4 *Cre*^−/−^, 9 *Cre*^+/−^). **f** Total plasma cholesterol in female mice was quantified. There was a significant effect of apoE isoform (F_2,41_ = 46.39, *p* < 0.0001) and Cre genotype (F_1,41_ = 24.31, *p* < 0.0001) with a significant interaction (F_2,41_ = 12.59, *p* < 0.0001). Post hoc pair-wise comparisons between *Cre*^+/−^ and *Cre*^−/−^ groups found *Cre* expression significantly increased plasma cholesterol levels in apoE2-expressing mice (*p* < 0.0001), but not apoE3 or apoE4-expressing mice. (n; E2F: 5 *Cre*^−/−^, 8 *Cre*^+/−^; E3F: 10 *Cre*^−/−^, 6 *Cre*^+/−^; E4F: 11 *Cre*^−/−^, 7 *Cre*^+/−^) **g** Total plasma triglycerides in male mice were quantified. There was a significant effect of apoE isoform (F_2,48_ = 13.31, *p* < 0.0001) and *Cre* expression (F_1,48_ = 36.70, *p* < 0.0001), with a significant interaction (F_2,48_ = 3.755, *p* = 0.0306). Post hoc pairwise comparisons between *Cre*^+/−^ and *Cre*^−/−^ groups found *Cre* expression significantly increased triglyceride levels in mice expressing apoE2 (*p* < 0.0001) or apoE3 (*p* = 0.0001), but not apoE4. (n; E2F: 8 *Cre*^−/−^, 11 *Cre*^+/−^; E3F: 11 *Cre*^−/−^, 12 *Cre*^+/−^; E4F: 4 *Cre*^−/−^, 9 *Cre*^+/−^) **h** Total plasma triglycerides in female mice were quantified. There was a significant effect of apoE isoform (F_2,40_ = 10.68, *p* = 0.0002) and *Cre* expression (F_1,40_ = 4.168, *p* = 0.0478), but no significant interaction (F_2,40_ = 0.1031, *p* = 0.9023). Post hoc pair-wise comparisons did not identify significant differences in triglyceride levels in mice dependent on *Cre* expression. (n; E2F: 4 *Cre*^−/−^, 8 *Cre*^+/−^; E3F: 10 *Cre*^−/−^, 6 *Cre*^+/−^; E4F: 11 *Cre*^−/−^, 7 *Cre*^+/−^) **i** Total plasma HDL in male mice was quantified. There was a significant effect of apoE isoform (F_2,49_ = 8.239, *p* = 0.0008), but not *Cre* expression (F_1,49_ = 0.9027, *p* = 0.3467) with a significant interaction (F_2,49_ = 5.434, *p* = 0.0074). (n; E2F: 8 *Cre*^−/−^, 12 *Cre*^+/−^; E3F: 11 *Cre*^−/−^, 12 *Cre*^+/−^; E4F: 4 *Cre*^−/−^, 9 *Cre*^+/−^). **j** Total plasma HDL in female mice was quantified. There was a significant effect of apoE isoform (F_2,42_ = 9.9098, *p* = 0.0005), but not *Cre* expression (F_1,42_ = 0.9699, *p* = 0.3304), with no interaction (F_2,42_ = 1.668, *p* = 0.2010). (n; E2F: 5 *Cre*^−/−^, 8 *Cre*^+/−^; E3F: 11 *Cre*^−/−^, 6 *Cre*^+/−^; E4F: 11 *Cre*^−/−^, 7 *Cre*^+/−^). Data analyzed by 2-way ANOVA followed by Holm-Sidak multiple comparisons testing. Values from EKO mice are also plotted according to sex (*n* = 5 males, 3 females), but were not included in statistical comparisons. * *p* < 0.05, ** *p* < 0.01, *** *p* < 0.001, **** *p* < 0.0001

While apoE is synthesized by a number of different organs outside of the CNS, the liver accounts for most circulating apoE [64]. Thus, we set out to ablate *APOE* expression in the liver through Cre-loxP technology, targeted through the hepatocyte-specific *Alb* promoter (that normally controls albumin expression). Specifically, mice expressing Cre-recombinase under the *Alb* promoter (*Alb-Cre* mice) [29] were crossed with *APOE-*KI mice for two successive generations to obtain *Alb-Cre* mice on all three human *APOE* backgrounds (*AlbCre*-EKI). The *AlbCre*-EKI mice were subsequently crossed with APP/PS1/EKI mice to achieve hepatocyte-specific knock-out of *APOE* in APP/PS1/EKI mice (APP/PS1/E2F^Cre^, APP/PS1/E3F^Cre^, APP/PS1/E4F^Cre^ mice, and APP/PS1/EKI^Cre^ mice collectively). Littermates lacking Cre-recombinase expression (Cre^−/−^) serve as controls, which are effectively APP/PS1/EKI mice. We first confirmed successful deletion of *APOE* from hepatocytes by performing qPCR analysis from liver tissue, which showed undetectable levels of *APOE* mRNA in Cre-expressing mice (Fig. 7a). We next measured apoE protein from liver tissue and also found the levels to be markedly decreased in Cre-expressing mice, regardless of *APOE* genotype (Fig. 7b). APP/PS1/E2F mice also had significantly more apoE protein in the liver relative to APP/PS1/E3F or APP/PS1/E4F mice, despite similar hepatic expression of *APOE* mRNA.

We also collected the plasma from APP/PS1/EKI^Cre^ mice and investigated the effect of *Alb-Cre* expression on the levels of apoE protein as well as the major lipid species. Due to the observed effect of sex on Aβ pathology in APP/PS1/EKI mice (Figs. 5 and 6), the males and females from this cohort were analyzed independently. Assessment of apoE levels in the plasma of male APP/PS1/EKI^Cre^ mice through an ELISA assay showed a significant effect of *APOE* genotype and *Cre* expression, with a significant interaction (Fig. 7c). Plasma apoE levels in female mice were also influenced by *APOE* genotype and Cre expression, with a significant interaction between the latter two parameters (Fig. 7d). We performed post hoc pair-wise comparisons between male *Cre*^+/−^ and *Cre*^−/−^ groups, and found *Cre* expression significantly decreased plasma apoE levels across apoE isoforms regardless of sex (Fig. 7c and d). Notably, residual amounts of apoE protein were detected in both male (Fig. 7c) and female (Fig. 7d) APP/PS1/E2F^Cre^ mice, likely because apoE2 has a low affinity for the LDL receptor.

As apoE plays an essential physiologic role in peripheral lipid homeostasis, we next examined the levels of total cholesterol (TC), triglycerides, and HDL in the plasma of APP/PS1/EKI^Cre^ mice. For these analyses, male and female were analyzed separately, and plasma samples from mice with global deletion of murine *Apoe* (EKO mice) were included for comparative purposes (*n* = 5 males, 3 females). Our analyses of male mice showed a significant effect of both *APOE* and *Cre* genotype on plasma TC, with a significant interaction between these two parameters. Post hoc pair-wise comparisons between *Cre*^+/−^ and *Cre*^−/−^ groups found *Cre* expression to significantly increase plasma TC levels in mice expressing apoE2 or apoE3, but not apoE4 (Fig. 7e). Similarly, significant effects of *APOE* and *Cre* genotype on plasma TC were found in female mice, with significant interaction between *APOE* and *Cre* genotype. Post hoc pair-wise comparisons between *Cre*^+/−^ and *Cre*^−/−^ groups found *Cre* expression to significantly increase plasma TC levels in apoE2-expressing mice, but not apoE3 or apoE4-expressing mice (Fig. 7f). In both male and female mice, the TC level in APP/PS1/E2F^Cre^ mice is of greatest magnitude, and appeared to be comparable to those found in EKO mice (though the EKO mice were not included in the direct statistical analysis). Plasma triglyceride concentrations in male mice follow a similar trend with a significant effect of *APOE* genotype and *Cre* expression, with a significant interaction. Post hoc pairwise comparisons between *Cre*^+/−^ and *Cre*^−/−^ groups found *Cre* expression significantly increased triglyceride levels in mice expressing apoE2 and apoE3, but not apoE4 (Fig. 7g). In female mice, there was a significant effect of *APOE* and *Cre* genotype, but no significant interaction. Post hoc pair-wise comparisons did not identify significant differences in triglyceride levels that are dependent on *Cre* expression (Fig. 7h). In male mice, there was a significant effect of *APOE* genotype, but not *Cre* expression, on plasma HDL levels, with a significant interaction between *APOE* and *Cre* genotypes (Fig. 7i). Similarly, there was a significant effect of *APOE* genotype, but not *Cre* expression in female mice (Fig. 7j). There were no significant interactions between *APOE* and *Cre* genotypes in female mice.

The changes in plasma lipid composition found in APP/PS1/EKI^Cre^ mice have some similarities and some differences compared to what was found in EKO mice. Though no direct statistical comparison was performed, the incongruence in some of the dataset relative to the EKO control could reflect the effect of residual extrahepatic *APOE* expression (e.g. in macrophages) that is below the detection limit of our ELISA assay.

### Liver-derived apoE does not influence Aβ accumulation in the brain

**Fig. 8:**
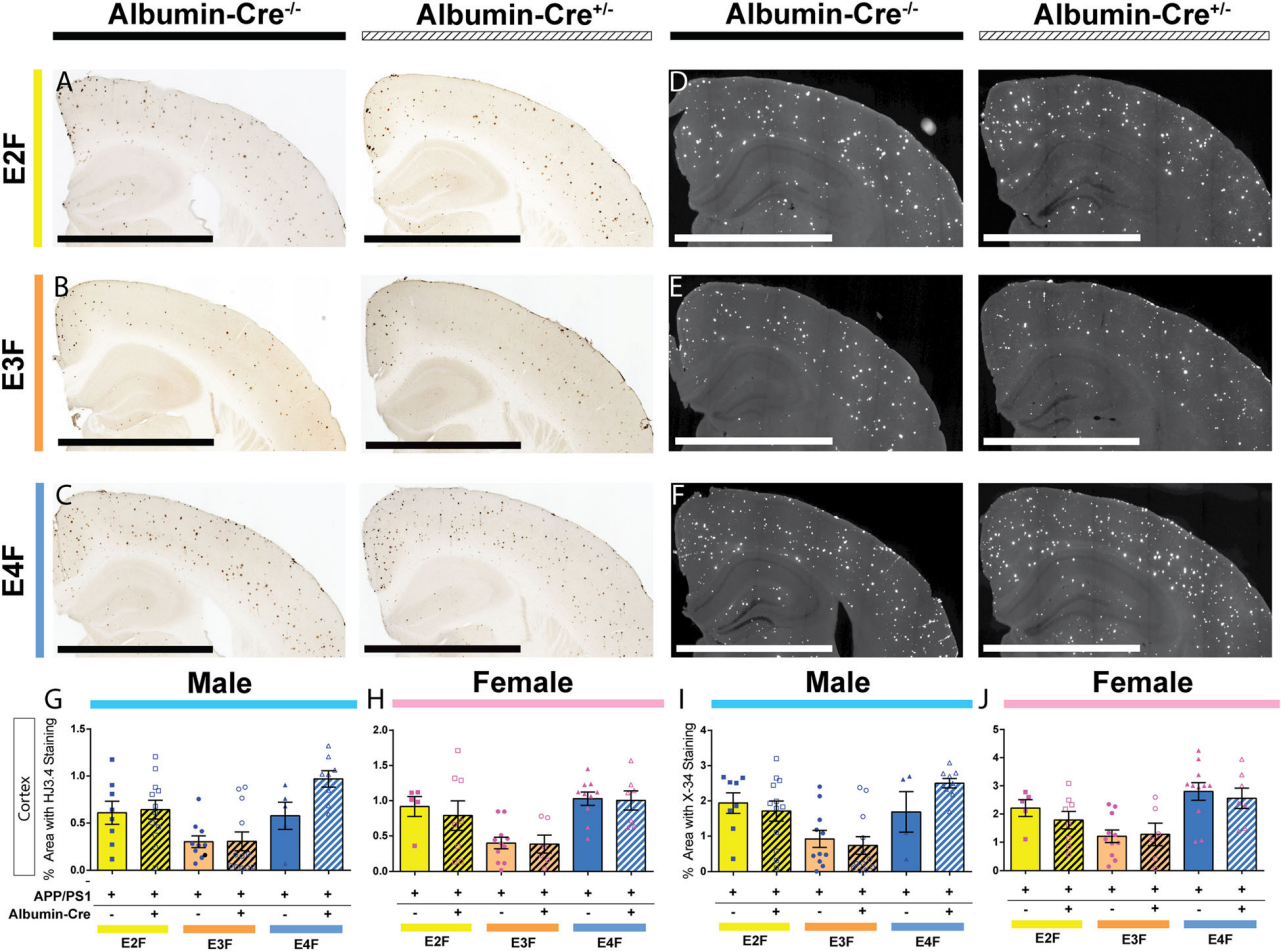
Hepatocyte-derived apoE does not influence Aβ accumulation in the brain. **a**, **b**, **c** Brain sections from 4-month-old APP/PS1/EKI^Cre^ mice and littermates were immunostained with an anti-Aβ antibody. Scale bars = 1 mm **g** The extent of cortical Aβ deposition in male mice was quantified. There was a significant effect of apoE isoform (F_2,49_ = 10.63, *p* = 0.0001), but not *Cre* expression (F_1,49_ = 2.693, *p* = 0.1072) with no interaction (F_2,49_ = 0.1845). (n; E2F: 8 *Cre*^−/−^, 12 *Cre*^+/−^; E3F: 10 *Cre*^−/−^, 12 *Cre*^+/−^; E4F: 5 *Cre*^−/−^, 8 *Cre*^+/−^). **h** The extent of cortical Aβ deposition in female mice was quantified. There was a significant effect of apoE isoform (F_2,41_ = 12.00, *p* < 0.0001), but not *Cre* expression (F_1,41_ = 0.2520, *p* = 0.6183) with no interaction (F_2,41_ = 0.09684, *p* = 0.9079). (n; E2F: 5 *Cre*^−/−^, 8 *Cre*^+/−^; E3F: 11 *Cre*^−/−^, 6 *Cre*^+/−^; E4F: 10 *Cre*^−/−^, 7 *Cre*^+/−^). **d**, **e**, **f** Brain sections from the same cohort were stained with X-34 dye. **i** The cortical fibrillar plaque load in male mice was quantified. There was a significant effect of apoE isoform (F_2,50_ = 11.34, *p* < 0.0001), but not *Cre* expression (F_1,50_ = 0.3027, *p* = 0.5846) with no interaction (F_2,50_ = 1.607, *p* = 0.2106). (n; E2F: 8 *Cre*^−/−^, 12 *Cre*^+/−^; E3F: 11 *Cre*^−/−^, 12 *Cre*^+/−^; E4F: 4 *Cre*^−/−^, 9 *Cre*^+/−^). **j** The cortical fibrillary plaque load in female mice was quantified. There was a significant effect of apoE isoform (F_2,42_ = 10.53, *p* = 0.0002), but not *Cre* expression (F_1,42_ = 0.5502, *p* = 0.4624) with no interaction (F_2,42_ = 0.2734, *p* = 0.7622). (n; E2F: 5 *Cre*^−/−^; 8 *Cre*^+/−^; E3F: 11 *Cre*^−/−^, 6 *Cre*^+/−^; E4F: 11 *Cre*^−/−^, 7 *Cre*^+/−^). Data analyzed by 2-way ANOVA

We next examined the effects of hepatocyte-specific *APOE* deletion on cerebral Aβ accumulation. Again, due to the observed effect of sex on Aβ pathology in APP/PS1/EKI mice (Figs. 5 and 6), the males and females from this cohort were analyzed independently. Brain sections from 4-month-old APP/PS1/EKI^Cre^ mice and littermate controls were assessed for Aβ immunostaining with HJ3.4b antibody (Fig. 8a, b, and c). Quantitative analyses in both male (Fig. 8g) and female (Fig. 8h) mice found a significant effect of *APOE* genotype, but not *Cre* expression on the cortical area covered by Aβ staining. To further characterize the nature of the deposited Aβ plaques, brain sections were stained with X-34 dye which only stains fibrillar plaques (Fig. 8d, e, and f). Quantitative analyses of the area covered by X-34 staining showed similar trends to those found in HJ3.4b staining, with a significant effect of *APOE* genotype, but not *Cre* expression, in both male (Fig. 8i) and female (Fig. 8j) mice.

**Fig. 9:**
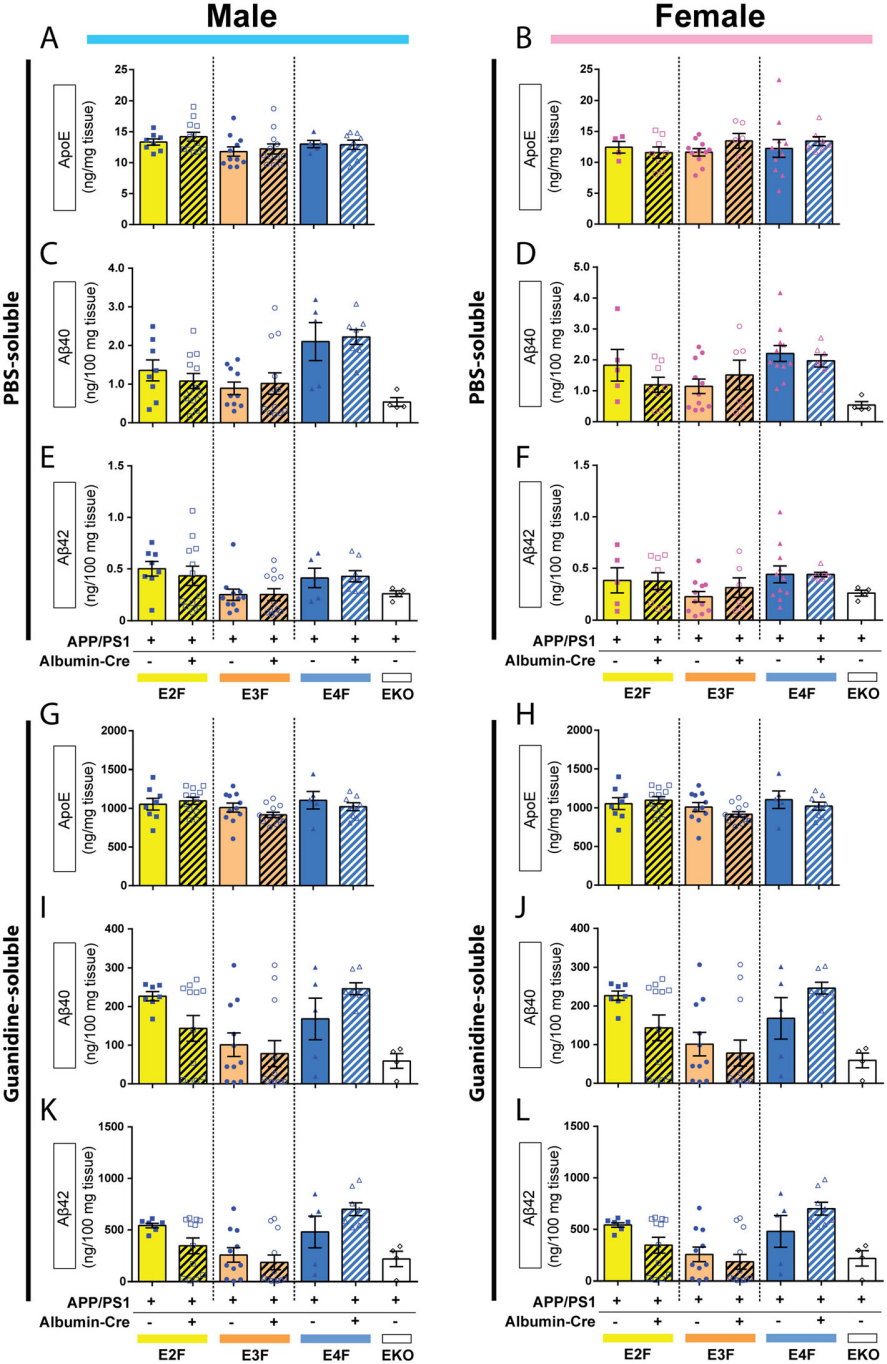
Hepatocyte-derived apoE does not affect apoE or Aβ levels in the brain. Cortical tissues were sequentially homogenized in PBS and 5 M guanidine HCl buffer. Aβ and apoE levels were quantified by ELISA. **a** Levels of PBS-soluble apoE in cortex of male mice. There were no significant effect of apoE isoform (F_2,50_ = 3.040, *p* = 0.568), *Cre* expression (F_1,50_ = 0.3673, *p* = 0.5472) or interaction (F_2,50_ = 0.1696, *p* = 0.8445). (n; APP/PS1/E2F: 8 *Cre*^−/−^,12 *Cre*^+/−^; APP/PS1/E3F: *Cre*^−/−^,12 *Cre*^+/−^; APP/PS1/E4F: 5 *Cre*^−/−^, 8 *Cre*^+/−^) **b** Levels of PBS-soluble apoE in cortex of female mice. There was no significant effect of apoE isoform (F_2,42_ = 0.1669, *p* = 0.8468), *Cre* expression (F_1,42_ = 0.02187, *p* = 0.8831) or interaction (F_2,42_ = 2.277, *p* = 0.1151). (n; APP/PS1/E2F: 5 *Cre*^−/−^, 8 *Cre*^+/−^; APP/PS1/E3F: 11 *Cre*^−/−^, 6 *Cre*^+/−^; APP/PS1/E4F: 11 *Cre*^−/−^, 7 *Cre*^+/−^) **c** Levels of PBS-soluble Aβ_40_ in the cortex of male mice. There was a significant effect of apoE isoform (F_2,49_ = 10.12, *p* = 0.0002), but not *Cre* expression (F_1,49_ = 0.002707, *p* = 0.9587) or interaction (F_2,49_ = 0.4113, *p* = 0.6650). (n; APP/PS1/E2F: 8 *Cre*^−/−^, 12 *Cre*^+/−^; APP/PS1/E3F: 10 *Cre*^−/−^, 12 *Cre*^+/−^; APP/PS1/E4F: 5 *Cre*^−/−^, 8 *Cre*^+/−^) **d** Levels of PBS-soluble Aβ_40_ in the cortex of female mice. There was a significant effect of apoE isoform (F_2,42_ = 3.495, *p* = 0.0394), but not *Cre* expression (F_1,42_ = 0.4314, *p* = 0.5149) or interaction (F_2,42_ = 1.217, *p* = 0.3065). (n; APP/PS1/E2F: 8 *Cre*^−/−^, 12 *Cre*^+/−^; APP/PS1/E3F: 11 *Cre*^−/−^, 12 *Cre*^+/−^; APP/PS1/E4F: 5 *Cre*^−/−^, 8 *Cre*^+/−^) **e** Levels of PBS-soluble Aβ_42_ in cortex of male mice. There was a significant effect of apoE isoform (F_2,50_ = 5.328, *p* = 0.0080), but not *Cre* expression (F_1,50_ = 0.07241, *p* = 0.7890), or interaction (F_2,50_ = 0.1759, *p* = 0.8392). (n; APP/PS1/E2F: 8 *Cre*^−/−^, 12 *Cre*^+/−^; APP/PS1/E3F: 11 *Cre*^−/−^, 12 *Cre*^+/−^; APP/PS1/E4F: 5 *Cre*^−/−^, 8 *Cre*^+/−^) **f** Levels of PBS-soluble Aβ_42_ in the cortex of female mice. There was a trend towards a significant effect of apoE isoform (F_2,42_ = 2.597, *p* = 0.0864), and no significant effect of Cre expression (F_1,42_ = 0.1586, *p* = 0.6925) or interaction (F_2,42_ = 0.2245, *p* = 0.7999). (n; APP/PS1/E2F: 5 *Cre*^−/−^, 8 *Cre*^+/−^; APP/PS1/E3F: 11 *Cre*^−/−^, 6 *Cre*^+/−^; APP/PS1/E4F: 11 *Cre*^−/−^, 7 *Cre*^+/−^) **g** Levels of guanidine-soluble apoE in the cortex of male mice. There was a trend towards a significant effect of apoE isoform (F_2,50_ = 2.513, *p* = 0.0912), and no effect of *Cre* expression (F_1,50_ = 0.8026, *p* = 0.3746) or interaction (F_2,50_ = 0.9035, *p* = 0.4116). (n; APP/PS1/E2F: 8 *Cre*^−/−^, 12 *Cre*^+/−^; APP/PS1/E3F: 11 *Cre*^−/−^, 12 *Cre*^+/−^; APP/PS1/E4F: 5 *Cre*^−/−^, 8 *Cre*^+/−^) **h** Levels of guanidine soluble apoE in the cortex of female mice. There was a significant effect of apoE isoform (F_2,42_ = 3.669, *p* = 0.0340), but no significant effect of *Cre* expression (F_1,42_ = 0.0003, *p* = 0.9857) or interaction (F_2,42_ = 0.2874, *p* = 0.7517). (n; APP/PS1/E2F: 5 *Cre*^−/−^, 8 *Cre*^+/−^; APP/PS1/E3F: 11 *Cre*^−/−^, 6 *Cre*^+/−^; APP/PS1/E4F: 11 *Cre*^−/−^, 7 *Cre*^+/−^) **i** Levels of guanidine-soluble Aβ_40_ in the cortex of male mice. There was a significant effect of apoE isoform (F_2,48_ = 7.273, *p* = 0.0017), but not *Cre* expression (F_1,48_ = 0.1102, *p* = 0.7414) or interaction (F_2,48_ = 2.333, *p* = 0.1079). (n; APP/PS1/E2F: 7 *Cre*^−/−^, 12 *Cre*^+/−^; APP/PS1/E3F: 11 *Cre*^−/−^, 12 *Cre*^+/−^; APP/PS1/E4F: 5 *Cre*^−/−^, 7 *Cre*^+/−^) **j** Levels of guanidine-soluble Aβ_40_ in the cortex of female mice. There was a significant effect of apoE isoform (F_2,41_ = 9.575, *p* = 0.0004), but no significant effect of *Cre* expression (F_1,41_ = 0.1030, *p* = 0.7499) and no interaction (F_2,41_ = 2.354, *p* = 0.1077). (n; APP/PS1/E2F: 4 *Cre*^−/−^, 8 *Cre*^+/−^; APP/PS1/E3F: 11 *Cre*^−/−^, 6 *Cre*^+/−^; APP/PS1/E4F: 11 *Cre*^−/−^, 7 *Cre*^+/−^) **k** Levels of guanidine-soluble Aβ_42_ in the cortex of male mice. There was a significant effect of apoE isoform (F_2,49_ = 10.88, *p* = 0.0001), but no significant effect of *Cre* expression (F_1,49_ = 0.05731, *p* = 0.8118), and a trend towards a significant interaction (F_2,49_ = 2.925, *p* = 0.0631). (n; APP/PS1/E2F: 7 *Cre*^−/−^, 12 *Cre*^+/−^; APP/PS1/E3F: 11 *Cre*^−/−^, 12 *Cre*^+/−^; APP/PS1/E4F: 5 *Cre*^−/−^, 8 *Cre*^+/−^). **l** Levels of guanidine-soluble Aβ_42_ in the cortex of female mice. There was a significant effect of apoE isoform (F_2,41_ = 16.32, *p* < 0.0001), but not *Cre* expression (F_1,41_ = 0.4266, *p* = 0.5173) and no interaction (F_2,41_ = 1.722, *p* = 0.1914). (n; APP/PS1/E2F: 4 *Cre*^−/−^, 8 *Cre*^+/−^; APP/PS1/E3F: 11 *Cre*^−/−^, 6 *Cre*^+/−^; APP/PS1/E4F: 11 *Cre*^−/−^, 7 *Cre*^+/−^). Data analyzed by 2 way ANOVA. Data from APP/PS1/EKO mice (3 males, 1 female) are also plotted, but were not included in statistical analysis

To examine whether *Alb-Cre* expression alters apoE protein levels in the brain of APP/PS1/EKI^Cre^ mice, we performed ELISA assays on cortical brain homogenates. In the PBS-soluble fraction from male mice, there was a trend towards a significant effect of *APOE* genotype, but no significant effect of *Cre* genotype, on apoE protein levels (Fig. 9a). In female mice, there was no significant effect of *APOE* or *Cre* genotype (Fig. 9b). No differences between any subgroups were detected on post hoc analyses of male or female mice. In the guanidine-soluble fraction from male mice, there was a trend towards a significant effect of *APOE* genotype, but not *Cre* genotype, on apoE protein level (Fig. 9g). In female mice, there was a significant effect of *APOE* genotype, but not *Cre* genotype, on apoE protein level (Fig. 9h). Post hoc analysis found no significant effect of *Alb-Cre* on guanidine-soluble apoE in either male (Fig. 9g) or female (Fig. 9h) mice.

Next, we analyzed total Aβ levels in APP/PS1/EKI^Cre^ mice and their respective *Cre*^−/−^ littermates. For these analyses, cortical samples from APP/PS1–21 mice with global deletion of murine *Apoe* (APP/PS1/EKO mice) were included for comparative purposes, and were not included in statistical analyses due to low n (*n* = 4, 3 males, 1 female). The cortices were sequentially homogenized in PBS and 5 M guanidine and the amounts of Aβ_40_ and Aβ_42_ were measured in each fraction via ELISA. In the PBS-soluble fraction from both male (Fig. 9c) and female (Fig. 9d) mice, we detected a significant effect of *APOE* genotype on the levels of Aβ_40_. Similarly, there was a significant effect of *APOE* genotype on Aβ_42_ levels in male mice (Fig. 9e). In female mice, there was a trend towards a significant effect of *APOE* genotype on the levels of Aβ42 (Fig. 9f). In our analyses of PBS-soluble Aβ_40_ or Aβ_42_, there were no significant effects of *Cre* expression or an interaction between *APOE* genotype and *Cre* expression in male or female mice. In the guanidine-soluble fraction, we also observed a significant effect of *APOE* genotype on Aβ_40_ (Fig. 9i) and Aβ_42_ (Fig. 9k) in male mice. In female mice, there was a significant effect of *APOE* genotype on Aβ_40_ (Fig. 9j) and Aβ_42_ (Fig. 9l) levels. In our analyses of guanidine-soluble Aβ_40_ or Aβ_42_, there were no significant effects of *Cre* expression or an interaction between *APOE* genotype and *Cre* expression in male or female mice. Of note, the APP/PS1/EKO mice had lower levels of Aβ_40_ or Aβ_42_ than most other genotypes, but direct comparison was not performed due to low *n* value.

Together, these results suggest that, while depletion of human *APOE* expression in hepatocytes led to a marked lowering of plasma apoE with some changes in plasma lipid composition, there were no significant effects on cerebral Aβ accumulation.

## Discussion

*APOE* is the strongest genetic risk factor for late-onset AD and intensive research efforts have led to several important insights regarding apoE and its role in AD. Nevertheless, cell-type specific roles for *APOE* isoform expression, secretion, and lipidation in neurodegenerative disease remain poorly understood. In an effort to help to begin to answer these and other questions, we created a new generation of human *APOE*-expressing mice to study cell-specific processes. Specifically, we generated three separate lines of *APOE*-KI mice, each carrying one of the three most common variants of the human *APOE* gene. The presence of loxP site on either side of the human gene sequence allow for cell-type-specific manipulation of *APOE* expression through the Cre-loxP system. Here, we characterized the newly created mice in terms of CNS as well as peripheral expression. We also qualitatively compared apoE particles isolated from astrocytes and microglia, and found the latter to produce significantly smaller lipid-containing particles. We took a step further to validate the functionality of the loxP sites by specifically ablating hepatocyte expression of *APOE*, effectively eliminating the majority of apoE protein in the plasma. We found this virtual absence of apoE in the plasma to cause significant alterations in the plasma lipid profile without significantly altering cerebral amyloid plaque accumulation in a model of Aβ-driven amyloidosis.

Our targeting construct retained the natural genetic context surrounding the human exon sequence, including endogenous regulatory elements such as enhancers. Thus, we expected the tissue-specific expression of the human *APOE* gene to closely parallel that of the endogenous mouse *Apoe* gene. Our *APOE* gene expression and protein analysis in the brain and plasma showed a similar expression level to wild type C57/BL6 mice and other *APOE* knock-in mice [1, 6, 43, 65]. Interestingly, we detected a subtle but statistically significant isoform-dependent difference in *APOE* mRNA levels in *APOE-*KI mice, where higher mRNA levels were detected in E4F mice relative to E2F or E3F mice in the hippocampus and lower levels of mRNA were detected in the brain hemisphere of E2F mice compared to E3F or E4F mice. These different mRNA levels could arise from compensatory changes in *APOE* transcription in response to isoform-dependent differences in protein stability or subtle differences in mRNA stability. Other than our new model, one other *APOE* knock-in model with floxed *APOE* alleles has been described [9]. In one experiment, apoE4 reduction in adult hippocampal astrocytes using an AAV-Cre vector resulted in a 50% decrease in insoluble Aβ_42_ in PDAPP mice [9]. Future studies using floxed allele APOE KI mice can test the temporal and cell-type specific effects of disrupting *APOE* expression on Aβ and tau pathology using a variety of inducible *Cre* mouse strains.

At 3 months of age, there was significantly less PBS-soluble apoE in the cortex of E4F mice compared to those from E2F or E3F mice at the same age, consistent with previously described findings in human and other *APOE* transgenic and knock-in mouse models [6, 37–44]. Concordantly, the level of apoE2 protein is the highest compared with other apoE isoforms in the CSF [6, 66], interstitial fluid (ISF) [67], brain parenchyma [68], and plasma [69–71]. The protein level of apoE2 is generally higher both in brain and plasma, likely due to decreased strongly reduced binding affinity to the LDL receptor [72]. Some studies report apoE4 is more susceptible to proteolysis compared to the other major isoforms of *APOE* [73, 74], and other studies have demonstrated the presence of apoE4 fragments (14–20 kDa) in AD brains [75, 76]. Structural differences between apoE3 and apoE4, particularly at the hinge region between the N- and C-terminal domains, may explain their different susceptibility to proteolytic degradation as well as lipid binding affinity [77]. In spite of these numerous observations, the exact nature of the protease responsible for apoE4 cleavage is unknown, although several candidates have been reported including cathepsin D [78], aspartic proteases [79], and a chymotrypsin-like protease [73]. A prior study using *APOE-*TR mice reported differences in apoE protein levels only in the hippocampus and cortex, regions that are susceptible to neurodegeneration in AD brains [43]. Significant differences in protein levels among apoE isoforms (apoE4 < apoE3 < apoE2) were also observed in the CSF and brain homogenates of *APOE-*TR mice when crossed to PDAPP mice [6]. It will be important in future studies to assess both CSF and brain levels of apoE in our new model in multiple brain regions as well as in all relevant cell types. Importantly, these results should be complemented with similar studies in human.

In regards to the effects of apoE isoforms in our new model on Aβ aggregation and accumulation in the brain, we found very similar findings that we described previously in APP/PS1–21 mice crossed to another *APOE* knock-in model [8, 10]. Namely, the presence of apoE4 in the brain resulted in significantly greater Aβ deposition than apoE3, similar to what is seen in humans [7, 80]. However, when different APP transgenic mouse strains were crossed with other *APOE* knock-in mice, apoE2-expressing mice generally exhibited less Aβ deposition than those expressing apoE3 or apoE4 [5, 6, 9], also similar to what is seen in humans. Crossing of APP/PS1–21 mice to our new *APOE*-KI model results in similar levels of Aβ accumulation in those expressing apoE2 and apoE4. Interestingly, crossing of another *APOE* knock-in model (*APOE-*TR mice) with APP/PS1–21 mice also resulted in similar levels of Aβ deposition in apoE2- and apoE4-expressing mice (Additional file 2: Figure S2a and S2c). The effect of sex on Aβ pathology in our new EKI models are intriguing, as we did not detect an effect of sex on Aβ pathology when the *APOE*-TR mice were crossed to the same strain of APP/PS1–21 mice (Additional file 2: Figure S2b and S2d). In another APP/PS1 transgenic model, 5XFAD mice, apoE2-expressing mice were also found to have similar Aβ deposition to those expressing apoE3 or apoE4 in the subiculum [43]. In aggressive amyloidogenic models such as APP/PS1–21 and 5XFAD, due to the particular *APP* and *PS1* mutations present, there is a much higher ratio of Aβ_42_ to other Aβ species than in many less aggressive APP transgenic mice [50]. Since Aβ_42_ is more aggregation-prone than other Aβ species [81], this difference might abrogate the effect of apoE2 on lowering Aβ pathology compared to other models and in humans. Further studies with our new model can test this hypothesis in other APP models such as APP knock-in mice or APP transgenic mice.

Outside of the brain, apoE is synthesized in multiple other sites, including the liver, spleen, adrenal gland, lung, testis, and ovary [82–84]. Total knock-out of *Apoe* results in severe hypercholesterolemia with accelerated atherosclerosis in the periphery [85, 86], accompanied in some [21] but not all studies [87] by synaptic loss and cognitive dysfunction. Due to the nature of a global knock-out, it was difficult to assess in the latter finding whether lack of apoE in the brain, in the periphery, or both, was responsible for the aforementioned phenotype. Given these outstanding questions, we tested whether specific ablation of a large source of peripheral apoE could modulate cerebral Aβ pathology. Indeed, virtual ablation of plasma apoE using an *Alb-Cre* line did not result in any change in cerebral apoE levels, which is consistent with previous reports on the inability of peripheral apoE to cross the BBB [20] or the blood-CSF barrier [88]. It is worth noting that hepatocytes are not the sole source of apoE in the periphery, as peripheral macrophages [82] and adipocytes [89] are known to contribute to the pool of apoE in the plasma. Accordingly, we did detect a non-negligible amount of apoE protein in plasma from APP/PS1/E2F^Cre^ mice, likely due to apoE2’s low affinity for the LDL receptor [90, 91] that results in slower clearance from the plasma. The lack of detection in APP/PS1/E3F^Cre^ and APP/PS1/E4F^Cre^ was perhaps due to a combination of lower protein levels and over-dilution that put the concentration outside of the assay’s detection limits. Our histological and biochemical analyses of cerebral Aβ pathology did not reveal a significant effect of knocking out liver-derived apoE at 4 months of age. Additionally, we failed to detect any changes in the degree of astrogliosis or microgliosis immediately surrounding the plaques between APP/PS1/EKI^Cre^ mice and their *Cre*^−/−^ littermates (data not shown). Altogether, our data suggest CNS- and peripherally-derived apoE exist in distinct pools that are independent from one another, as has been suggested in human studies [92]. However, we cannot rule out other hypotheses that would otherwise explain the lack of any appreciable effect on Aβ in APP/PS1/EKI^Cre^ mice, such as an unknown adaptive response that masked the contribution of hepatic apoE to brain Aβ pathology.

Alternatively, it is possible that apoE from one pool can indirectly exert an effect on the other side of the BBB. For example, a recent study showed that restoration of peripheral *Apoe* expression led to a partial rescue of cognitive phenotypes in mice lacking apoE in the brain [28]. These findings suggest a dual mechanism by which apoE deficiency causes behavioral deficits, and that peripherally derived apoE may influence neuronal function through an indirect mechanism, such as vascular dysfunction secondary to dyslipidemia or via effects on brain endothelial cells that make up the BBB. In support of this latter hypothesis, prior studies found the BBB in EKO mice to be severely compromised [24, 93], and the severity (or permeability of the BBB) also increased with age [93]. Thus, it is conceivable that an effect of hepatocyte-derived apoE on brain Aβ pathology may be observed in aged APP/PS1/EKI^Cre^ mice, or if conditional knock-out of hepatic apoE occurs at a later stage of Aβ pathology. Such an effect may be modulated by apoE’s effects on cerebrovascular function and/or through its ability to cross a leaky BBB. Some researchers have proposed a two-hit vascular hypothesis of AD cerebrovascular damage, where hit 1 is an initial insult on the BBB itself that is sufficient to initiate neuronal injury and neurodegeneration, but can also promote accumulation of Aβ in the brain through defects in clearance of Aβ through the BBB (reviewed extensively in [94, 95]).

BBB dysfunction is a common co-occurrence and may directly contribute to neurodegeneration and cognitive decline in AD [94, 95]. In support of this latter hypothesis, data obtained from an older *APOE* knock-in model also support an isoform-dependent effect of apoE on BBB integrity, where EKO and apoE4-expressing mice developed vascular defects before neuronal and synaptic changes occur [25]. A more recent study showed apoE4-expressing mice to have impaired spontaneous BBB repair following traumatic brain injury (TBI) compared to apoE2- or apoE3-expressing mice [96]. Both studies explored various mechanisms that could explain for the negative influence of apoE4 on the BBB integrity, all of which involved changes in astrocytes and pericytes located in the neurovascular unit. However, it is theoretically possible that peripherally derived apoE species can somehow contribute to this process, especially with the leakiness of the BBB in early stages of TBI and later stages of AD. As there are several sources of apoE in the periphery, they could differentially affect BBB homeostasis. Intriguingly, a prior study utilized bone marrow transplant to show that BBB homeostasis depends on equal contributions from tissue and blood cell derived apoE, but lack of *Apoe* expression in bone marrow-derived cells alone was enough to significantly increase the BBB permeability [93]. These findings underscore the putative role for leukocytes (e.g. macrophages) in BBB maintenance. It remains elusive how apoE from bone marrow-derived cells may contribute to the integrity of the BBB, the dysfunction of which had been proposed to contribute to neurodegeneration in AD [95]. In this context, it would be interesting to investigate whether restoration of *APOE-ε4* expression in peripheral macrophages could rescue BBB defects in EKO mice, and how that compares to restoration of *APOE-ε2* or *APOE-ε3* expression.

It is our hope that these new *APOE*-KI mice will facilitate studies into apoE physiology and AD pathogenesis. It is also important, however, to acknowledge their limitations. While the *APOE*-KI mice harbor the human gene sequence, they retain the regulatory elements found in mice. Considerable species differences between rodents and humans exist and might challenge our ability to generate findings that are all relevant and directly translatable to humans from studies in mice and rats. Apparent differences in physiological function and metabolism, such as lipid metabolism and immune response between humans and rodents might preclude some discoveries that are relevant to disease mechanism. For example, apoB is a ligand for the LDLR along with apoE in humans, albeit with a lower affinity than apoE. Hepatic-derived apoB is secreted as apoB100 (a full length protein) and contains the LDLR binding domain. However, a large portion of hepatically derived apoB in mice is truncated (apoB48) and does not contain the LDLR domain. Wild-type mouse VLDL and IDL contain roughly equal portions of apoB48 and apoB100, and this leads to a compromised compensatory mechanism in the absence of apoE, leading to severe hypercholesterolemia in *Apoe* knock-out mice [86, 97]. This latter example highlights the need to address these and other caveats when interpreting rodent studies, especially in those where such physiologic differences might confound some findings.

## Conclusions

We employed a targeted gene replacement strategy to generate mouse lines that express the three most common alleles of the human *APOE* gene at physiological levels. We fully characterized all three lines with respect to their apoE levels in the CNS as well as the plasma, from early life to adulthood. We partially recapitulated the isoform-dependent effect of *APOE* on Aβ accumulation in a model of amyloidosis. Furthermore, we also validated the functionality of the loxP sites in facilitating constitutive, tissue-specific, knock-out of *APOE* through liver-specific expression of Cre-recombinase. Lastly, we also investigated the effects of knocking out liver-derived apoE on plasma lipid profiles and Aβ deposition in the brain. Moving forward, these mice should prove an invaluable asset for further studies on the physiological and pathophysiological roles of *APOE*, especially the role of apoE isoforms in specific cell types and different organs in the context of AD and other neurodegenerative diseases.

## Additional files

Additional file 1: **Figure S1.** Replacement of the mouse *Apoe* gene with the human *APOE* gene in *APOE-*KI mice. **a** The specific sequence of the genomic region surrounding the translation initiation codon of the human and mouse *APOE* gene, located on exon 2, is shown. The mouse and human sequences are aligned for comparative purposes. The cleavable signal peptide is encoded within exons 2 and 3 (amino acids 1–18). **b, c, d** Targeting vectors for the ε2 and ε4 alleles have identical sequence other than their respective SNPs at positions 130 and 176, both located on exon 4 ([Cys130, Cys176] for *APOE-ε2* ***(B)***, [Cys130, Arg176] for *APOE-ε3* (**c**), and [Arg130, Arg176] for *APOE-ε4*) (**d**). The red arrowheads identify the SNPs at positions 130 and 176 in each of the allele sequences. **e, f** *APOE* mRNA levels in the hippocampus (**e**), and hemisphere (**f**) of *APOE-*KI mice were analyzed at 3 months of age (*p* = 0.0169, F = 5.843 and *p* = 0.0025, F = 10.88, respectively). **g** Cortex-derived brain homogenates from 3-month-old *APOE-*KI mice were subjected to western blot analysis for apoE using antibody HJ15.3. White arrowhead = sialylated apoE (MW ~ 35.3 kDa). Black arrow = non-sialylated apoE (MW ~ 33.6 kDa). *p < 0.05, **p < 0.01,***p < 0.001. A one-way ANOVA was used to assess significance between more than two groups, and Bonferroni’s post-hoc test was used to test for differences between each of the groups. All values are reported as mean ± SEM. *N* = 5 per genotype for ELISA analysis, with approximately equal numbers of males and females. (TIF 3191 kb)

Additional file 2: **Figure S2.** ApoE isoforms differentially influence Aβ plaque deposition in APP/PS1/APOE-TR mice. **a** Brain sections from 3-month-old APP/PS1/APOE-TR mice were immunostained with anti-Aβ antibody 3D6 and the extent of Aβ deposition in the cortex quantified. Two-way ANOVA analysis found a significant effect of apoE isoform (F_2,38_ = 10.13, *p* = 0.0003) but no significant effect of sex (F_1,38_ = 0.001177, *p* = 0.9728), and no interaction (F_2,38_ = 0.06101, *p* = 0.9409). Post hoc analysis comparing apoE isoform within each sex found a statistically significant increase in Aβ deposition in male apoE2- (*p* = 0.0010) and apoE4-expressing (*p* = 0.0430) mice compared to apoE3, APP/PS1/E2 = 8 males, 9 female; APP/PS1/E3 = 11 males, 4 females; APP/PS1/E4 = 7 males, 5 females.**b** Since there was no effect of sex on Aβ staining, data for both males and females were pooled together. A one-way ANOVA was performed (F = 11.92, *p* < 0.0001), followed by Tukey’s post hoc test for multiple comparisons. **c** Brain sections from 4-month-old APP/PS1/APOE-TR mice were stained with X-34 dye that recognizes only fibrillar plaques and the cortical area stained by X-34 was quantified. There was a significant effect of genotype (F_2,37_ = 8.701, *p* = 0.0008) but no significant effect of sex (F_1,37_ = 0.6014, *p* = 0.4430), and no interaction (F_2,37_ = 0.2230, *p* = 0.8012). Post hoc analysis comparing apoE isoform within each sex found a statistically significant increase in X-34 staining in male apoE2-expressing mice compared to apoE3 (*p* = 0.0011), APP/PS1/E2 = 8 males, 9 female; APP/PS1/E3 = 10 males, 4 females; APP/PS1/E4 = 7 males, 5 females. **d** Since there was no effect of sex on X-34 staining, data for both males and females were pooled together. A one-way ANOVA was performed (F = 11.69, *p* = 0.0001), followed by Tukey’s post hoc test for multiple comparisons. * *p* < 0.05, ** *p* < 0.01, **** *p* < 0.0001. All values are reported as mean ± SEM. (TIF 605 kb)

## Data Availability

The datasets used and/or analysed during the current study are available from the corresponding author on reasonable request.

## Abbreviations

AD: Alzheimer disease
ApoE: Apolipoprotein E
Aβ: Amyloid-β
BAC: Bacterial artificial chromosome
EKO: ApoE knock-out
GFAP: Glial fibrillary acidic protein
HDL: High density lipoprotein
IBA1: Ionized calcium binding adaptor molecule 1
LDL(R): Low density lipoprotein (receptor)
NDGGE: Non-denaturing gradient gel electrophoresis
SEM: Standard error of the mean
TC: Total cholesterol
